# Mapping and Validation of Stem Rust Resistance Loci in Spring Wheat Line CI 14275

**DOI:** 10.3389/fpls.2020.609659

**Published:** 2021-01-12

**Authors:** Zennah C. Kosgey, Erena A. Edae, Ruth Dill-Macky, Yue Jin, Worku Denbel Bulbula, Ashenafi Gemechu, Godwin Macharia, Sridhar Bhavani, Mandeep S. Randhawa, Matthew N. Rouse

**Affiliations:** ^1^Kenya Agricultural and Livestock Research Organization, Njoro, Kenya; ^2^Department of Plant Pathology, University of Minnesota, Saint Paul, MN, United States; ^3^Cereal Disease Laboratory, United States Department of Agriculture-Agricultural Research Service, Saint Paul, MN, United States; ^4^Debre Zeit Agricultural Research Center, Ethiopian Institute of Agricultural Research, Bishoftu, Ethiopia; ^5^International Maize and Wheat Improvement Center (CIMMYT), Texcoco, Mexico; ^6^CIMMYT - World Agroforestry Centre (ICRAF), Nairobi, Kenya

**Keywords:** *Triticum aestivum*, *Puccinia graminis* f. sp. *tritici*, genetics, QTL, disease resistance, stem rust, Ug99

## Abstract

Stem rust caused by *Puccinia graminis* f. sp. *tritici* (*Pgt*) remains a constraint to wheat production in East Africa. In this study, we characterized the genetics of stem rust resistance, identified QTLs, and described markers associated with stem rust resistance in the spring wheat line CI 14275. The 113 recombinant inbred lines, together with their parents, were evaluated at the seedling stage against *Pgt* races TTKSK, TRTTF, TPMKC, TTTTF, and RTQQC. Screening for resistance to *Pgt* races in the field was undertaken in Kenya, Ethiopia, and the United States in 2016, 2017, and 2018. One gene conferred seedling resistance to race TTTTF, likely *Sr7a*. Three QTL were identified that conferred field resistance. QTL *QSr.cdl-2BS.2*, that conferred resistance in Kenya and Ethiopia, was validated, and the marker Excalibur_c7963_1722 was shown to have potential to select for this QTL in marker-assisted selection. The QTL *QSr.cdl-3B.2* is likely *Sr12*, and *QSr.cdl-6A* appears to be a new QTL. This is the first study to both detect and validate an adult plant stem rust resistance QTL on chromosome arm 2BS. The combination of field QTL *QSr.cdl-2BS.2*, *QSr.cdl-3B.2*, and *QSr.cdl-6A* has the potential to be used in wheat breeding to improve stem rust resistance of wheat varieties.

## Introduction

Wheat accounts for about 20% of global human consumed calories and proteins with hexaploid wheat [*Triticum aestivum* (2*n* = 6*x* = 42; AABBDD)] being the most widely cultivated species (Gupta et al., 2008). The European Union, China, India, Russia, and the United States are the top five wheat producers (Food and Agriculture Organization [FAO], 2018). Production of wheat is challenged by both abiotic and biotic factors. Among biotic constraints, stem rust of wheat caused by *Puccinia graminis* f. sp. *tritici* (*Pgt*) is considered one of the most devastating fungal diseases with the potential to result in a 100% yield loss on susceptible varieties. The *Pgt* race Ug99, first isolated from Uganda in the year 1999 (Pretorius et al., 2000) and later characterized as race TTKSK (Jin et al., 2008), was the first *Pgt* race virulent to stem rust resistance gene *Sr31* (Pretorius et al., 2000; Wanyera et al., 2006). The unique virulence combination in race TTKSK rendered more than 90% of global wheat varieties susceptible during testing in 2005 (Singh et al., 2006). Since then, multiple derivatives of TTKSK have been reported, including TTKST, virulent to *Sr24* and identified in 2006 (Jin et al., 2008); TTTSK, virulent to *Sr36* and identified in 2007 (Jin et al., 2009); and TTKTT, virulent to *SrTmp* and identified in 2014 (Newcomb et al., 2016; Patpour et al., 2016). Host resistance remains the most effective and economical approach to controlling stem rust, and this can be expressed at the seedling stage and at the adult plant stage (Priyamvada et al., 2011). Plants with major-effect resistance genes (also known as R-genes) may express intermediate to immune levels of resistance when challenged by an avirulent race or strain of the pathogen. Some of the major genes currently utilized in breeding programs to confer resistance to *Pgt* are *Sr13*, *Sr22*, *Sr25*, *Sr26*, *Sr33*, and *Sr50* (Helguera et al., 2003; Mago et al., 2005, 2013, 2015; Simons et al., 2011; Periyanan et al., 2013; Singh et al., 2015). Genes with temporary designations, such as *SrND643* and *SrNing*, are also being utilized to provide resistance against *Pgt* (Lopez-Vera et al., 2014; Basnet et al., 2015). The downfall of deploying major-effect genes individually is that they can be easily overcome by the pathogen, resulting in what is referred to as “boom-and-bust” cycles. The Ug99 race group clearly highlights the boom-and-bust cycle by the defeat of important R-genes, such as *Sr24*, *Sr31*, *Sr36*, and *SrTmp* that were widely deployed worldwide (Pretorius et al., 2000; Jin et al., 2008, 2009).

The adult plant resistance genes, such as *Sr2* (likely pleiotropic with *Yr30*), *Sr55* (pleiotropic with *Lr67*, *Yr46*, *Pm46*), *Sr56*, *Sr57* (pleiotropic with *Lr34*, *Yr18*, *Pm38*), and *Sr58* (pleiotropic with *Lr46*, *Yr29*, *Pm39*), confer slow rusting resistance at the adult plant stage with each having a minor resistance effect and, thus, possibly not providing adequate resistance when present alone under high disease pressure (Bansal et al., 2014; Herrera-Foessel et al., 2014; Singh et al., 2015). Combinations of effective major-effect race-specific resistance genes (R-genes) and/or minor-effect race non-specific resistance genes may result in durable resistance, or resistance that remains effective for a longer period of time (Parlevliet and van Ommeren, 1988; Priyamvada et al., 2011). Identification and deployment of new resistance genes into advanced wheat lines is necessary for new resistance gene combinations to be assembled.

CI 14275 is a Canadian breeding line (Q-2331-34) that was a part of the 1966 USDA-ARS International Spring Wheat Rust Nursery and that appeared effective to the Ug99 race group in Kenya when screened in 2005, 2006, and 2007 (Rouse et al., 2011). The USDA National Small Grains Collection lists the pedigree of CI 14275 as Thatcher^∗^6/Kenya Farmer//6^∗^Lee/Kenya Farmer. Thatcher has been reported to have *Sr5*, *Sr9g*, *Sr12*, and *Sr16* genes (McIntosh et al., 1995). The effectiveness of resistance in Thatcher to stem rust at the adult plant stage is shown to involve *Sr12* (Rouse et al., 2014b) and can be enhanced when combined with *Sr57* (*Lr34*) (Kolmer et al., 2011). Kenya Farmer has *Sr6*, *Sr7a*, *Sr9b*, *Sr10*, *Sr11*, and *Sr12* genes, whereas Lee has *Sr5*, *Sr9g*, *Sr11*, *Sr12*, and *Sr16* genes (Knott, 1957; McIntosh et al., 1995). CI 14275 previously exhibited low infection types to multiple races with broad virulence at the seedling stage (Rouse et al., 2011). In the field, CI 14275 was highly resistant to a composite of races in St. Paul, Minnesota, in addition to being moderately resistant to the Ug99 race group in Kenya (Rouse et al., 2011). The objectives of this study were to characterize the genetics of stem rust resistance in the spring wheat line CI 14275, identify QTLs associated with the resistance, and develop markers linked to the identified resistance QTL.

## Materials and Methods

### Development of the Primary Population

One hundred thirteen recombinant inbred lines (RILs) (*F*_7__:__9_) were developed from a cross between LMPG-6 [a stem rust–susceptible spring wheat line (Knott, 1990)] and CI 14275 (LMPG-6/CI 14275) through single-seed descent at the USDA-ARS Cereal Disease Laboratory (CDL), Minnesota in the United States.

### Development of the Validation Population

From the 113 RILs (*F*_7__:__9)_ derived from LMPG-6/CI 14275, line #162 was selected because it expressed a consistent level of resistance to stem rust across the three environments: Ethiopia (0-20MSS), Kenya (0-15SMS), and St. Paul, Minnesota (5RMR-25RMR). Additionally, line #162 was among the early maturing lines. Line #162 was crossed with Kwale, a susceptible Kenyan cultivar, to generate 180 *F*_3__:__4_ families (Kwale/Line #162).

### Phenotyping of the Primary Population in the Greenhouse

The LMPG-6/CI 14275 RIL population, together with the parents, were evaluated for their seedling infection types to *Pgt* races TTKSK (isolate 04KEN156/04; Ug99) and TRTTF (06YEM34-1) in a Biosafety level 3 (BSL-3) greenhouse and for infection types to *Pgt* US races TPMKC (74MN1409), TTTTF (01MN84A-1-2), and RTQQC (04MN74-1) at the USDA-ARS CDL greenhouse in Minnesota, following an inoculation method described by Rouse et al. (2011). Square plastic pots (6 cm × 6 cm × 6 cm) were filled with 100% vermiculite. Five seeds for each of four lines per pot were planted in two replicates and placed in a greenhouse with temperature and light conditions as described previously (Hundie et al., 2019). *Pgt* isolates were derived from single pustules, increased in isolation and stored at −80°C. When primary leaves were fully emerged, the inoculum was prepared by removing *Pgt* urediniospores from −80°C, heat shocking for 15 min in a 45°C water bath, rehydrating for approximately 2–4 h in a chamber maintained at 80% relative humidity by a KOH solution, and suspending in lightweight mineral oil (Soltrol 70; ConocoPhillips Inc., Houston). The plants were inoculated by spraying them with a suspension using an atomizer. After inoculation, the plants were then placed in a fume hood for 30 min to allow the oil to evaporate and, thereafter, transferred to a dew chamber at 100% relative humidity for 14–16 h. After the dew chamber incubation, plants were returned to the greenhouse bench and maintained at 18 ± 2°C with a photoperiod of 16 h.

Two weeks postinoculation, seedlings were assessed for seedling infection types (ITs) based on the 0–4 scale developed by Stakman et al. (1962). The description of infection types used to classify the reactions to *Pgt* are as follows: ‘0’ = no uredinia or any other sign of infection, ‘;’ = no uredinia but presence of hypersensitive necrotic flecks, ‘1’ = small uredinia surrounded by yellow chlorotic or necrotic areas, ‘2’ = small- to medium-sized uredinia in a dark green island surrounded by a chlorotic area, ‘3’ = medium-sized uredinia, surrounded by light green chlorosis, and ‘4’ = large uredinia with no or a limited amount of chlorosis. All observed infection types on the same leaf were recorded with the infection type(s) listed in order according to their prevalence. A comma (,) symbol was used to separate multiple ITs observed on the same plant with the most frequent IT recorded first. Whenever multiple infection types were observed on different plants of the same line, a forward slash (/) symbol was used to separate the infection types. A letter ‘C’ was recorded when extensive chlorosis was associated with the infection. The plus (+) and minus (−) symbols were used for the pustules that were relatively larger or smaller, respectively, than normally associated with a given IT. Plants with ITs ranging from 0 to 2 were categorized as resistant, and those ranging from 3 to 4 were categorized as susceptible. The seedling infection types assigned on a 0–4 scale were then converted to a 0–9 linear scale according to Gao et al. (2019) for use in analyses. We calculated the raw mean values of the linearized infection types for each pathogen race across the two replications for use in subsequent QTL analyses. RILs with mean linearized values of 6 or less were considered resistant, whereas those with values greater than 6 were categorized as susceptible.

### Phenotyping of the Primary Population in the Field

The experimental lines were evaluated for stem rust severity in two African field locations: Njoro, Kenya, and Debre Zeit, Ethiopia, in 2016, 2017, and 2018. Evaluations in Africa took place during the off-season of approximately January through May. The lines were evaluated in St. Paul, Minnesota, in 2017 and 2018. Across the three locations, approximately 5 g seed was sown in each of two replicates planted in a randomized complete block design. In Njoro, Kenya, the field plots were two 0.7-m-long rows separated by a distance of 0.3 m. Disease spreader rows consisting of a mixture of susceptible cultivars Cacuke, Eagle 10, Robin, and six CIMMYT lines carrying *Sr24* were planted to surround the entries 10–14 days before the experimental plots were planted. The disease spreader row mixture was also planted as hill plots on one side of the experimental plots to facilitate the buildup and spread of the disease. From the booting to heading growth stages (Zadok’s growth stages 37–60), freshly collected urediniospores of locally predominant Ug99 *Pgt* races TTKSK, TTKST, TTKTK, and TTKTT were bulked and suspended in distilled water, and approximately 1 mL of the suspension was injected into disease spreader row plants using a hypodermic syringe. Inoculations were conducted in the afternoons over 3 occasions with 7-day intervals. In Debre Zeit, Ethiopia, the experimental plots were planted as double 1-m-long rows, and disease spreader rows, including cultivars Arendeto, Digalu, Local Red, Morocco, and PBW343, were planted perpendicular to the plots and were artificially inoculated at Zadok’s growth stage 37–60 with bulked urediniospores from predominant *Pgt* races TTKSK, TKTTF, TRTTF, and JRCQC to initiate infection on the plots. In St. Paul, the experimental plots were single 1-m-long rows, separated by a distance of 0.3 m. The spreader rows that consisted of cultivars Baart, Morocco, and Thatcher were planted 1–2 weeks earlier than entries and sown perpendicular to the experimental plots. At the heading stage, the plots were spray-inoculated using an Ulva+ sprayer (Micron Sprayers Ltd., Bromyard, United Kingdom) with a light mineral oil suspension of bulked urediniospores of North American *Pgt* races QFCSC (isolate 06ND76C), TPMKC (isolate 74MN1409), RKRQC (isolate 99KS76A), RCRSC (isolate 77ND82A), QTHJC (isolate 75ND717C), and MCCFC (isolate 59KS19). Disease on the inoculated spreader rows initiated stem rust infection on the experimental plots.

When the spreaders had attained 50% severity in the three locations, stem rust severity was visually scored in the experimental plots based on the modified Cobb scale of 0–100, where 0 = immunity (no uredinia or any other sign of infection) and 100% = completely susceptible (Peterson et al., 1948). Infection response was rated as resistant (R), small uredinia surrounded by necrosis; moderately resistant (MR), medium-sized uredinia surrounded by necrosis or chlorosis; moderately susceptible (MS), medium-sized uredinia without necrosis; susceptible (S), large uredinia without necrosis; or MRMS, an infection response that included both the MR and MS categories (Roelfs et al., 1992). Coefficient of infection (COI) values were generated by multiplying the stem rust severity value for each line by a constant value for each infection response: 0 = 0, R = 0.2, RMR = 0.3, MR = 0.4, M = 0.6, MS = 0.8, S = 1.0 (Knott, 1989). Average coefficient of infection for the two replicates were determined and used for analyses. Raw mean coefficients of infection values across the two replicates were used for QTL analyses.

### Phenotyping of the Validation Population in the Field

Twice-replicated plots of the 180 *F*_3__:__4_ validation lines were evaluated for field response to the Ug99 race group in Njoro, Kenya, in 2018. Planting; inoculation with *Pgt* races that included TTKSK, TTKST, TTKTK, and TTKTT; and evaluation was conducted as previously described.

### DNA Extraction

Tissues were harvested from three-leaf-stage single plants of the RIL population (*F*_7__:__9_), from single F_3_ plants of the Kwale/line #162 population, and their parents into Eppendorf tubes (1.5 mL). The harvested leaves were dried and ground for 1–2 min using a Genogrinder (SPEX Sample Prep). Extraction buffer (300 μl: 200 mM Tris-HCL pH 8.0, 250 mM NaCl, 25 mM EDTA, 0.5% SDS, ddH_2_0) was added to each well, the tubes were shaken gently before incubating at room temperature for 15 min, followed by centrifuging completed at 2500 rpm. Working under a fume hood, 300 μl of chloroform:isoamyl alcohol (24:1) was added to each well, the suspension was mixed, plates were centrifuged for 20 min at 2500 rpm, and the supernatant (200–300 μl) was transferred to separate tubes. Cold isopropanol (300 μl) was then added to each tube. Plates were shaken gently and placed in a refrigerator for 10–20 min. After decanting the supernatant, 300 μl of 70% ethanol was added to each tube, the tubes were centrifuged for 20 min at 2,500 rpm, the ethanol was poured out, and the DNA pellets were air-dried. When the pellets were completely dry, 100 μl of distilled H_2_O was added to each tube to resuspend the pellet and the tubes were left in a refrigerator overnight. DNA was quantified using a NanoDrop 1000 spectrophotometer (Thermo Fisher, Waltham, MA, United States), and concentrations were diluted to 50 ng/μl.

### Genotyping of the Primary Population

Genotyping of the *F*_7__:__9_ RILs and their parents was conducted at the USDA-ARS small grains genotyping lab in Fargo, North Dakota, using the iSelect 90k SNP assay developed by Wang et al. (2014). The 90k SNP assay contains 81,587 SNPs and was developed for allohexaploid and allotetraploid wheat populations. The raw 90K SNP data together with the consensus map were uploaded onto Illumina GenomeStudio genotyping software version 2.0. The SNPs were manually called and the genotypes grouped as AA, AB, or BB. The missing genotypes were indicated as no call (NC). Following calculation of allele frequencies, a total of 12,243 polymorphic markers with both AA and BB allele frequency greater than 0.25 were selected.

### QTL Mapping

The allele calls from GenomeStudio were used to create genetic linkage maps with the MSTMap algorithm (Wu et al., 2008) implemented in ASMap R package (Taylor and Butler, 2017) with the following parameters: distance function Kosambi, cut_off_p_value set at 1e-08, no_map_dist set at 15, no_map_size set at 0, missing_threshold set at 0.10, detect_bad_data set at “yes” and objective function set at “COUNT.” A total of 33 linkage groups, each with greater than five markers were generated. The 33 linkage groups included a total of 819 markers (Supplementary Table 1). The genotype calls were manually curated by coding single-marker, double-crossover events as NC. In addition, unusual heterozygous calls were manually curated by replacing these calls with NC. These 33 linkage groups were used for QTL analysis using the R/qtl software (Broman and Sen, 2009). The QTL that conferred resistance to races RTQQC, TTTTF, and TPMKC at the seedling stage and QTL that conferred resistance at the adult plant stage in the Kenya, Ethiopia, and United States environments were identified separately. Interval mapping was conducted using the scanone function, and single-QTL genome scan via Haley-Knott regression was performed. The statistical significance thresholds of the genome scan results were determined by running 1000 permutation tests with a 0.05 significance level. QTL were also reported when LOD values were greater than 3.0, and the corresponding markers were detected in multiple environments. Markers flanking the QTL were identified using the find.flanking function. Linkage groups were assigned to chromosomes based on the 90K consensus map. The physical positions of the QTL were identified by searching positions of the flanking markers in the T3/Wheat website: https://triticeaetoolbox.org/wheat/ (Blake et al., 2016). The probe sequence of the markers was determined by searching the GrainGenes website, https://wheat.pw.usda.gov/GG3/, when marker information was missing in the T3/Wheat website, and the sequences were blasted to the reference genome at the BLAST website: https://wheat-urgi.versailles.inra.fr/Seq-Repository/BLAST to find the physical position of the markers (Alaux et al., 2018).

### Confirmation of Presence/Absence of *Lr34*/*Sr57* in CI 14275

The marker csLV34 (Lagudah et al., 2006) linked to the pleiotropic rust resistance gene *Lr34/Sr57/Yr18* was genotyped on the parents (LMPG-6 and CI 14275). Wheat cultivar Chinese Spring was used as the positive control for the gene.

### Data Analyses

Chi-squared analyses were performed on the linearized seedling data to determine goodness-of-fit to the expected segregation ratios for different inheritance models. Using R software, analysis of variance (ANOVA) was performed using the average COI values with lines and environments both as fixed variables and replication at random. Correlation of stem rust severity between the environments was determined using Pearson correlation coefficients.

## Results

### Phenotyping in the Greenhouse

One hundred thirteen *F*_7__:__9_ RILs and their parents showed susceptible responses when tested against *Pgt* races TTKSK and TRTTF in two replicates each (Supplementary Table 2). A 33+ IT was observed on the resistant parent, CI 14275, and an IT of 3/33+ was observed on the susceptible parent, LMPG-6, when tested with race TTKSK (Table 1 and Supplementary Table 2). ITs of 33+ and 3+/33+ were observed on the parents CI 14275 and LMPG-6, respectively, when tested with race TRTTF (Table 1 and Supplementary Table 2). On the other hand, ITs of; 1/1+ 3C and 3+ were observed on the resistant and susceptible parents, respectively, when tested with race TTTTF, and the observed frequency of the population fit into the expected ratio of 1:1 (one gene) with a χ^2^ value of 0.704 (*p* = 0.40) (Table 1). Screening for race RTQQC resulted in 3/33+ and 3+/33+ ITs on CI 14275 and LMPG-6, respectively. Nearly all of the RILs were susceptible to RTQQC in at least one replication, and the rare low responses to RTQQC were usually not consistent across the two replications (Supplementary Table 2 and Table 1). For race TPMKC, ITs of 23−/23+ and 3+/33+ were observed on the resistant and susceptible parents, respectively, and the observed frequency of the population did not fit any simple expected ratio (Supplementary Table 2 and Table 1).

**TABLE 1:** Segregation of stem rust resistance in 113 recombinant inbred lines (RILs) along with response of parents of the cross LMPG-6/CI 14275 against five *Puccinia graminis* f. sp. *tritici* races.

**Races**	**Number of Lines**		**Seedling Infection Types^a^**		**Chi-square**	***P*-value**	**Number of segregating genes^b^**
	**Resistant**	**Susceptible**	**Susceptible parent – LMPG-6**	**Resistant parent – CI 14275**			
RTQQC	0	113	3+/33+	3/33+	–	–	None
TTTTF	57	56	3+	;1/1 + 3C	0.704	0.40	One
TPMKC	16	97	3+/3+3	23−/23+	–	–	Unknown
TRTTF	0	113	3+/33+	33+	–	–	None
TTKSK	0	113	3/33+	33+	–	–	None

*^*a*^Infection types (ITs) follow the Stakman et al. (1962) scale. The plus (+) and minus (−) symbols are used for the pustules that were relatively larger and smaller, respectively, than normal; a forward slash (/) symbol separates multiple infection types observed on different plants of the same line; fleck (;) indicates no uredinia but presence of hypersensitive necrotic flecks; ITs ranging from 0 to 2 were categorized as resistant, and ITs ranging from 3 to 4 were categorized as susceptible; the dash (–) symbol indicates no generated data. ^*b*^Unknown, segregation of RILs did not fit any expected ratio; none, no resistant plants.*

### Phenotyping in the Field

**FIGURE 1:**
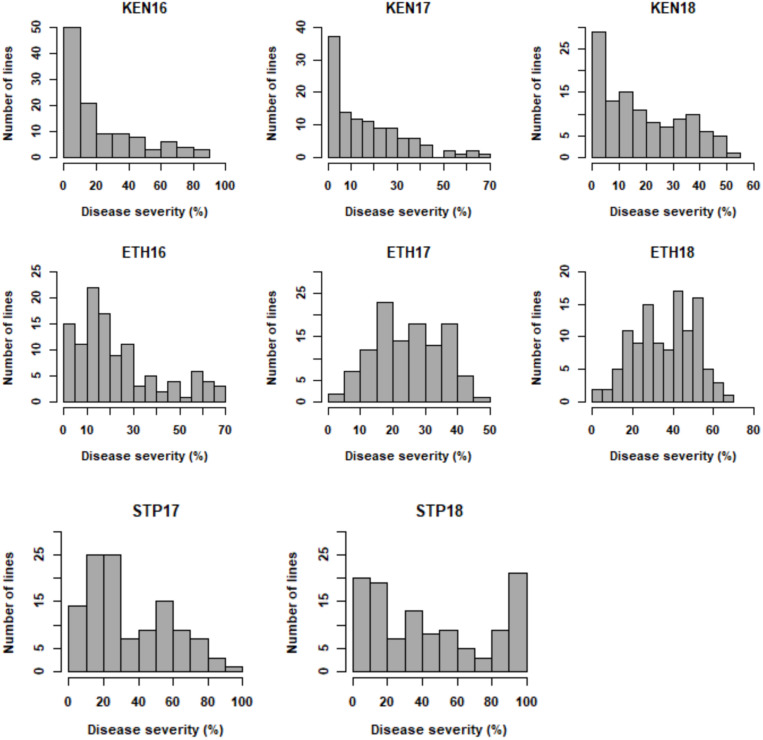
Frequency distribution for stem rust severity (%) in RILs developed from the cross LMPG-6/CI 14275 evaluated in Kenya (KEN), Ethiopia (ETH), and St. Paul (STP) in 2016, 2017, and 2018.

For the primary population, stem rust ratings from 0 to 90S were observed in Kenya 2016 (KEN16), 0 to 80S in Kenya 2017 (KEN17), and TR (trace R) to 60S in Kenya 2018 (KEN18) (Supplementary Table 3). Ratings from TR to 80S were observed in Ethiopia 2016 (ETH16), TMS (trace MS) to 50S in Ethiopia 2017 (ETH17), and TMR (trace MR) to 70S in Ethiopia 2018 (ETH18) (Supplementary Table 4). Ratings from 5RMR to 100S were observed in St. Paul 2017 (STP17) and TR to 80S in St. Paul 2018 (STP18) (Supplementary Table 5). Stem rust ratings from 0 to 70S were observed in the validation population. In the three Kenyan environments: KEN16, KEN17, and KEN18, the distribution of stem rust severity was somewhat skewed toward resistance (Figure 1). A symmetric distribution was observed in the ETH16, ETH17, and ETH18 environments (Figure 1). A somewhat symmetric distribution was observed in the STP17 and STP18 environments (Figure 1). ANOVA indicated significant effects for lines and environment, a significant interaction between the RILs and the environments, and a non-significant effect for replication (Table 2). Stem rust severity over the different environments had statistically significant correlation coefficients, ranging between 0.55 and 0.85 for correlations among all environments except with ETH17, with which the correlations were low but still statistically significant, ranging between 0.21 and 0.47 (Table 3).

**TABLE 2:** Analysis of variance for stem rust severity of 113 recombinant inbred lines (RILs) and the parents of the cross LMPG-6/CI 14275 tested in eight environments (Kenya – 2016, 2017, and 2018; Ethiopia – 2016, 2017, and 2018; St. Paul – 2017 and 2018).

**Source**	**Degrees of freedom**	**Mean square**	***F*-value^a^**
Lines	114	3653.2	30.7***
Environments	7	24,129.2	202.6***
Replication	1	215.2	1.8
Lines × Environments	786	486.0	4.1***
Error	876	119.1	

^a^***Significant at 0.001 probability level.

**TABLE 3:** Correlation coefficients among stem rust coefficient of infection (COI) values for 113 recombinant inbred lines (RILs) and the parents of the cross LMPG-6/CI 14275 in eight environments (Kenya – 2016, 2017, and 2018; Ethiopia – 2016, 2017, and 2018; St. Paul – 2017 and 2018)^a^.

**Environment**	**KEN16**	**ETH16**	**STP17**	**KEN17**	**ETH17**	**STP18**	**KEN18**	**ETH18**
KEN16	1.00							
ETH16	0.82	1.00						
STP17	0.57	0.63	1.00					
KEN17	0.73	0.73	0.59	1.00				
ETH17	0.25	0.21	0.30	0.32	1.00			
STP18	0.64	0.69	0.85	0.65	0.25	1.00		
KEN18	0.72	0.74	0.55	0.78	0.42	0.62	1.00	
ETH18	0.67	0.73	0.57	0.67	0.47	0.64	0.73	1.00

*^*a*^KEN16, Njoro, Kenya 2016; KEN17, Njoro, Kenya 2017; KEN18, Njoro, Kenya 2018; ETH16, Debre Zeit, Ethiopia 2016; ETH17, Debre Zeit, Ethiopia 2017; ETH18, Debre Zeit, Ethiopia 2018; STP17, St. Paul, MN 2017; STP18, St. Paul, MN 2018.*

### Confirmation of Presence/Absence of *Lr34*/*Sr57* in CI 14275

Both parents were negative for the positive allele of the *Lr34* gene-based marker, indicating that the resistance in CI 14275 did not involve *Lr34*/*Sr57*. This result was confirmed by the lack of significant QTL on chromosome 7D, where *Lr34* is located.

### LMPG-6/CI 14275 Genetic Map

The genetic map of LMPG-6/CI 14275 is listed in Supplementary Table 1. A total of 819 markers mapped to 33 linkage groups. The 33 linkage groups corresponded to 15 wheat chromosomes. Six wheat chromosomes were missing from the genetic map: 2D, 3A, 4B, 4D, 6D, and 7D. The remaining wheat chromosomes were represented in the map from anywhere between one and four linkage groups.

### Identified QTLs at Seedling and Adult Plant Stages

The 0.05 LOD significance thresholds for each trait ranged from 3.63 to 3.81. We did not detect any significant QTL for response of the primary population to RTQQC, TPMKC, or ETH17. All significant QTL are reported in Table 4 in addition to QTL with an LOD of 3.0 or greater that were detected in multiple environments.

**TABLE 4:** Quantitative trait loci (QTL) identified in the recombinant inbred lines (RILs) population derived from the cross LMPG-6/CI 14275 for seedling resistance to *Puccinia graminis* f. sp. *tritici* races RTQQC, TPMKC, and TTTTF and for field resistance in Njoro, Kenya (2016, 2017, and 2018); Debre-Zeit, Ethiopia (2016, 2017, and 2018); and St. Paul, Minnesota (2017 and 2018).

**Race/environment^a^**	**QTL^b^**	**Donor of resistant allele**	**Linkage Group^c^**	**LOD^d^**	**Position (cM)^e^**	**Left flanking marker^f^**	**Right flanking marker^g^**	**Phenotypic variance (%)^h^**
**Seedling stage**								
TTTTF	*QSr.cdl-4AL*	CI 14275	4AL_2	18.4	1	Tdurum_contig42019_1714	BS00009680_51	42.3
**Adult plant stage**								
KEN16	*QSr.cdl-2BS.2*	CI 14275	2B	3.1	0	Tdurum_contig54704_176	BS00038820_51	14.7
	*QSr.cdl-3B.2*	CI 14275	3B	3.6	177	Excalibur_c57658_54	IAAV3838	16.4
KEN17	QSr.cdl-3B.2	CI 14275	3B	6.7	177	Excalibur_c57658_54	IAAV3838	18.3
KEN18	*QSr.cdl-3B.2*	CI 14275	3B	5.9	177	Excalibur_c57658_54	IAAV3838	21.5
ETH16	*QSr.cdl-6A*	CI 14275	6A	3.8	103	Excalibur_c60006_452	BS00023627_51	12.6
	QSr.cdl-2BS.2	CI 14275	2B	5.25	0	Tdurum_contig54704_176	BS00038820_51	17.6
ETH18	*QSr.cdl-6A*	CI 14275	6A	3.49	106	BS00023627_51	IAAV3806	8.7
	*QSr.cdl-2BS.2*	CI 14275	2B	4.73	0	Tdurum_contig54704_176	BS00038820_51	10.5
	QSr.cdl-4AL	CI 14275	4AL_2	3.0	3	Tdurum_contig42019_1714	BS00009680_51	4.7
STP16	*QSr.cdl-6A*	CI 14275	6A	3.0	103	Excalibur_c60006_452	BS00023627_51	6.4
STP17	*QSr.cdl-3B.2*	CI 14275	3B	5.1	194	RAC875_c10595_473	RAC875_c69_499	14.0
	*QSr.cdl-6A*	CI 14275	6A	3.1	104	Excalibur_c60006_452	BS00023627_51	7.1
STP18	*QSr.cdl-3B.2*	CI 14275	3B	4.2	193	RAC875_c10595_473	RAC875_c69_499	15.8

*^*a*^Abbreviations of experimental locations: KEN16, Njoro, Kenya 2016; KEN17, Njoro, Kenya 2017; KEN18, Njoro, Kenya 2018; ETH16, Debre Zeit, Ethiopia 2016; ETH18, Debre Zeit, Ethiopia 2018; STP17, St. Paul, MN 2017; STP18, St. Paul, MN 2018. ^*b*^Naming of QTL: Q, QTL; Sr, stem rust; cdl, cereal disease lab. ^*c*^Linkage groups as listed in Supplementary Table 1. ^*d*^LOD, logarithm of odds value. ^*e*^cM, centiMorgan, position of the peak LOD. ^*f*^Left flanking marker, markers flanking the identified QTL, on the left side. ^*g*^Right flanking marker = markers flanking the identified QTL, on the right side. ^*h*^Percent of variance explained by the identified QTL.*

### QTL on Chromosome Arm 2BS

One QTL mapped to chromosome arm 2BS in the ETH16 and ETH18 environments and was designated *QSr.cdl-2BS.2* (Table 4). This QTL also reached an LOD of 3.1 in KEN16. The QTL is flanked by the markers Tdurum_contig54704_176 and BS00038820_51 (Table 4). We designated this QTL with the suffix *2BS.2* because *QSr.cdl-2BS* was already designated (Rouse et al., 2014b). The phenotypic variance explained by the QTL ranged from 10.5 to 17.6% across the three environments (Table 4).

### QTL on Chromosome 3B

One QTL mapped to chromosome 3B in the KEN17, KEN18, STP17, and STP18 environments and was designated *QSr.cdl-3B.2* (Table 4). This QTL also reached an LOD of 3.6 in KEN16. We designated this QTL with the suffix *3B.2* because *QSr.cdl-3B* was already designated (Rouse et al., 2014b). The markers Excalibur_c57658_54, IAAV3838, RAC875_c10595_473, and RAC875_c69_499 were linked to *QSr.cdl-3B.2* (Table 4). The phenotypic variance explained by the QTL in the five environments ranged from 14.0 to 21.5% (Table 4).

### QTL on Chromosome Arm 4AL

One QTL mapped to chromosome arm 4AL for race TTTTF and was designated *QSr.cdl-4AL* (Table 4). The QTL *QSr.cdl-4AL* was flanked by markers Tdurum_contig42019_1714 and BS00009680_51 (Table 4). This QTL explained a large proportion (42.3%) of the phenotypic variance observed (Table 4). Interestingly, this QTL was observed in the field in ETH18 with an LOD of 3.0 (Table 4).

### QTL on Chromosome Arm 6A

One QTL mapped to chromosome arm 6A in the ETH16, ETH18, STP16, and STP17 environments with LOD values of 3.8, 3.5, 3.0, and 3.1, respectively. This QTL did not pass the 0.05 LOD threshold in any of these environments, but it was detected above LOD 3.0 in four environments, which adds confidence to the validity of this QTL. We designated the QTL as *QSr.cdl-6A* (Table 4). The markers BS00023627_51, IAAV3806, and Excalibur_c60006_452 were linked to *QSr.cdl-6A* (Table 4). The phenotypic variance explained by the QTL in the four environments tested ranged from 6.4 to 12.6% (Table 4).

### Combined 2BS, 3B, and 6A QTLs

Three QTLs, *QSr.cdl-2BS.2, QSr.cdl-3B.2*, and *QSr.cdl-6A*, were identified only at the adult plant stage in seven of the eight field environments. No QTL were reported in ETH17 because disease pressure was low although the line responses in ETH17 were significantly correlated with those in other environments. Based on the genotype data of the QTL peak markers, seven lines were found to have all three QTLs combined, and 11 lines lacked the three QTLs. The *t*-test results from the two groups (with and without the three QTLs) showed statistically significant differences (*p* < 0.01; 0.001) in seven environments (all environments except ETH17) (Table 5). The combined QTL provided large reductions in stem rust severity in the observed environments and appeared to be a highly effective combination even when the disease pressure was high (Table 5).

**TABLE 5:** Coefficient of infection (COI) (%) for LMPG-6/CI 14275 lines with and without three adult plant resistance (APR) quantitative trait loci (QTL): *QSr.cdl-2BS.2*, *QSr.cdl-3B.2*, and *QSr.cdl-6A* detected in six environments tested in this study, excluding ETH17.

**Environment^a^**	**KEN16**	**KEN17**	**KEN18**	**KEN mean**	**ETH16**	**ETH18**	**ETH mean**	**STP17**	**STP18**	**STP mean**	**Overall mean**
Mean value of seven lines without QTL	60.2	36.6	35.5	43.5	54.5	51.6	52.3	65.3	92.9	79.1	56.7
Average Mean value for of 11 lines with combination of three QTL detected	6.7	3.7	6.9	5.9	8.8	14.7	12.4	23.8	19.1	21.4	12.3
*P*-value^b^	**	**	***	***	***	***	***	***	***	***	***

^*a*^Abbreviations of experimental locations: KEN16, Njoro, Kenya 2016; KEN17, Njoro, Kenya 2017; KEN18, Njoro, Kenya 2018; ETH16, Debre Zeit, Ethiopia 2016; ETH18, Debre Zeit, Ethiopia 2018; STP17, St. Paul, MN 2017; STP18, St. Paul, MN 2018. ^b^**Significant at p < 0.01 probability level, ***significant at p < 0.001 probability level.

### Kompetitive Allele-Specific PCR (KASP) Markers

KASP assay primers were designed corresponding to 15 90K SNP markers linked to QTL, but only six of these KASP assays produced clear polymorphism in the validation population (Table 6). Simple ANOVAs for each marker were independently run to determine whether any of the KASP markers predicted the phenotype in the validation population. One marker linked to *QSr.cdl-2BS.2*, Excalibur_c7963_1722_C1, was associated with reduced stem rust severity in Njoro, Kenya, in 2018 (*p*-value = 0.003). This validated *QSr.cdl-2BS.2*, which was identified in the LMPG-6/CI 14275 population in the KEN16, ETH16, and ETH18 environments. Two KASP assays derived from markers linked to *QSr.cdl-6A* were polymorphic but were not associated with stem rust severity. We then discovered that line #162 did not possess the resistant alleles for markers linked to *QSr.cdl-6A* after reviewing the original 90K allele calls. Line #162 did possess the resistant alleles of markers linked to *QSr.cdl-2BS.2* and *QSr.cdl-3B.2.* Line #162 was selected only based on phenotype, which explains the absence of *QSr.cdl-6A.* The observed polymorphism in the two KASP assays derived from markers linked to *QSr.cdl-6A* may be explained by broken linkage between the resistant allele of these markers and *QSr.cdl-6A* in the susceptible parent of the validation population: Kwale. Only one of the KASP assays that we designed for markers on chromosome 3B was polymorphic: Tdurum_contig32277_121. This marker was not associated with stem rust severity in the validation population. This raised the possibility that both parents of the validation population were fixed for the presence of *QSr.cdl-3B.2*. Because we considered *QSr.cdl-3B.2* as likely conferred by *Sr12*, we tested the validation population with the *Sr12* marker from Hiebert et al. (2016), NB-LRR-3, and found that the parents and population were fixed for presence of the *Sr12*-associated allele. Two additional KASP assays were designed based on markers linked to *QSr.cdl-4AL*. These assays were not associated with stem rust severity in Kenya, where the pathogen population is virulent to *Sr7a*.

**TABLE 6:** Primer sequences of polymorphic Kompetitive allele-specific PCR (KASP) assays in the line #162/Kwale population derived from 90K single nucleotide polymorphism (SNP) markers linked to identified quantitative trait loci (QTL)^a^.

**SNP**	**A1**	**A2**	**C1**	**SNP1**	**SNP2**
Tdurum_contig32277_121	GAAGGTGACCAAGTTCATGCTGG TGGCATATGAATGATATGACTTCT	GAAGGTCGGAGTCAACGGATTGG TGGCATATGAATGATATGACTTCG	CGCGTGTACTCTA CCAAATCCAACAA	A	C
Excalibur_c7963_1722	GAAGGTGACCAAGTTCATGCTGG GGGCATATGAATGATATGACTTCT	GAAGGTCGGAGTCAACGGATTGG TGGCATATGAATGATATGACTTCG	CGCGTGTACTCTA CCAAATCCAACAA	A	C
Ra_c6672_1679	GAAGGTGACCAAGTTCATGCTGG TGGCATATGAATGATATGACTTCT	GAAGGTCGGAGTCAACGGATTGG TGGCATATGAATGATATGACTTCG	CGCGTGTACTCTA CCAAATCCAACAA	A	C
Jagger_c8310_70	GAAGGTGACCAAGTTCATGCTGG TGGCATATGAATGATATGACTTCT	GAAGGTCGGAGTCAACGGATTGG TGGCATATGAATGATATGACTTCG	CGCGTGTACTCTA CCAAATCCAACAA	A	C
IAAV3806	GAAGGTGACCAAGTTCATGCTAGT TCAAGATACCACAGCAACAACAAA	GAAGGTCGGAGTCAACGGATTGT TCAAGATACCACAGCAACAACAAC	GTTTCTGGGCGTG GTGTTTCTTTGTA	T	G
BS00031178_51	GAAGGTGACCAAGTTCATGCTAATAG TTTCTAGAAAATATGTTGTCTCCTTTT	GAAGGTCGGAGTCAACGGATTAGT TTCTAGAAAATATGTTGTCTCCTTTC	TGAGGTTGAAGGAA CTAACTTTTTAATGCT	A	G

* ^*a*^SNP markers linked to the identified QTL; A1, A2, and C1 are the primers.*

## Discussion

The RIL population, together with the parents, was susceptible at the seedling stage when tested with *Pgt* races TTKSK, TRTTF, and RTQQC. This is an indication that the resistant parent, CI 14275, lacked an effective major gene conferring resistance to these races. This observation is different from that observed by Rouse et al. (2011) for the seedling response of CI 14275 to race TTKSK. Different environmental conditions between the two studies, particularly temperature (Zhang et al., 2017; Chen et al., 2018; Gao et al., 2019), may explain the variable responses although the differences in these findings may warrant further investigation. Similarly, the observed inconsistencies of infection types between replicates in some lines could have been due to the temperature effect on segregating stem rust resistance genes. The seedling resistance to race TTTTF was conferred by a single gene. Based on map location and race specificity, this gene is likely *Sr7a* (Turner et al., 2016; Saini et al., 2018). The distribution of disease severities for the RILs at the adult plant stage across all the environments was mostly continuous but somewhat skewed toward resistance in the Kenyan environments, suggesting a quantitative type of resistance. Lower correlation coefficients (0.21–0.32) were observed between ETH17 and the ETH16, KEN16, KEN17, STP17, and STP18 environments, suggesting genotype by environment interactions as indicated by the ANOVA results (Table 2). Stem rust severities of the entire population were relatively low in the ETH17 environment. The differences in environmental conditions, different races used for inoculation, and the amount of inoculum used in different environments could have also contributed to the observed significant genotype by environment interactions.

The identified QTL *QSr.cdl-2BS.2* on chromosome arm 2BS with the explained phenotypic variance ranging from 10.5% to 17.6%, conferred adult plant resistance in the KEN16, ETH16, and ETH18 environments. The QTL was also identified in the validation population in Kenya in 2018. Several stem rust resistance genes have been mapped on chromosome arms 2BS and 2BL. The stem rust resistance genes effective to race TTKSK that mapped to chromosome arm 2BS are *Sr36*, *Sr39*, and *Sr40* (Wu et al., 2009; Niu et al., 2011; Rouse et al., 2012). The gene *Sr36* was identified in *Triticum timopheevi*, *Sr39* was identified in *Aegilops speltoides*, and *Sr40* was identified in *Triticum araraticum*. The resistance provided by the *Sr36* gene was overcome by race TTTSK (virulent to *Sr31* and *Sr36*) (Jin et al., 2009), whereas *Sr39* and *Sr40* genes appear effective against the Ug99 race group but have not been widely utilized in breeding programs due to their potential linkage drag. The alien species origin of genes *Sr39* and *Sr40* as well as susceptible ITs observed in the seedlings of the entire RIL population along with parents against race TTKSK ruled out the possibility of *Sr39* or *Sr40* conferring the observed resistance in this study.

The mapped genes on chromosome arm 2BL are *Sr9h*, *Sr16*, *Sr28*, and *Sr47* (Tsilo et al., 2007; Hiebert et al., 2011; Rouse et al., 2014a). The genes *Sr9h*, *Sr16*, and *Sr28* were identified in *Triticum aestivum*, whereas the gene *Sr47* was identified in *A. speltoides*. The *Sr16* gene was shown to be ineffective against Ug99 races (Jin et al., 2007). Because *Sr16* and *QSr.cdl-2BS.2* are on different chromosome arms, they cannot be the same. The *Sr28* and *Sr47* genes have been found effective against the Ug99 race group (Jin et al., 2007; Rouse et al., 2012), but the QTL *QSr.cdl-2BS.2* identified in this study is unlikely either the *Sr28* or *Sr47* gene because of the observed susceptibility of CI 14275 at the seedling stage in response to race TTKSK. The *Sr9* alleles *Sr9a*, *Sr9b*, *Sr9d*, *Sr9e*, *Sr9f*, and *Sr9g* are ineffective against Ug99 isolates, but the *Sr9h* allele has been found effective against race TTKSK and other races in the Ug99 race group with virulence to *Sr31* (Rouse et al., 2014a). Based on the physical position of the peak marker, the *QSr.cdl-2BS.2* found in this study is located around 58.3 Mb on the 90K consensus map (Wang et al., 2014). This map position excludes the possibility that *QSr.cdl-2BS.2* could be conferred by stem rust resistance genes on the long arm of chromosome 2B. The *QSr.umn-2B.2* reported by Bajgain et al. (2015) in a RB07/MN06113-8 population is between 22.7 and 40.7 Mb on 2B, and *QSr.cdl-2BS* reported by Rouse et al. (2014b) in the Thatcher/McNeal population is located between 12.0 and 31.7 Mb on 2B. The physical location of the reported *QSr.cdl-2BS.2* is near these two previously reported QTL. Rouse et al. (2014b) found that the resistant allele of the reported *QSr.cdl-2BS* was contributed by Thatcher. Because Thatcher is a major component of the pedigree of CI 14275, it is likely that *QSr.cdl-2BS* from Rouse et al. (2014b) and *QSr.cdl-2BS.2* from this study are conferred by the same gene(s) derived from Thatcher. Tdurum_contig54704_176, the peak marker associated with *QSr.cdl-2BS.2*, is located at 61 cM in the popseq consensus map, whereas the Excalibur_c7963_1722 marker that was picked up in the validation population is located at 60 cM in the popseq consensus map. A KASP marker was developed for Excalibur_c7963_1722 because it was found to be a flanking marker of *QSr.cdl-2BS.2* in a preliminary QTL analysis. Several other studies have reported stem rust resistance QTLs on chromosome 2B (association mapping study QTL at 79.6 Mb on 2BL: Letta et al., 2013; GWAS study QTL located between 11.3 and 79.9 Mb: Prins et al., 2016; Cacuke/Huhwa and Cacuke/Yaye population between 65.8 and 73.9 Mb on 2BL: Randhawa et al., 2018). Our study is the first to both detect and validate an adult plant stem rust resistance QTL on chromosome arm 2BS.

*QSr.cdl-3B.2* explains between 14.0 and 21.5% of the phenotypic variance and confers adult plant resistance in the KEN16, KEN17, KEN18, STP17, and STP18 environments. *QSr.cdl-3B.2* is flanked by markers Excalibur_c57658_54, IAAV3838, RAC875_c10595_473, and RAC875_c69_499 in the various environments. The *Sr2* gene is located on chromosome arm 3BS, whereas *Sr12* is located near the centromere on chromosome 3B. Both were derived from *Triticum turgidum*. *Sr2* is an adult plant resistance gene used in breeding as a source of durable resistance to stem rust although the gene may not provide adequate resistance under high disease pressure. *Sr12* is present in all of the parents of CI 14275, cultivars Thatcher, Kenya Farmer, and Lee; therefore, *Sr12* is almost certainly present in CI 14275 (unless there is a mistake in the pedigree or the gene postulations of the parents). The identified *QSr.cdl-3B.2* in this study is located between 58.6 and 75.9 Mb on 3B and overlaps other reported QTLs on 3B (Thatcher/McNeal population located between 15.4 and 85.8 Mb, Rouse et al., 2014b; association mapping population located between 13.8 and 59.5 Mb, Bajgain et al., 2015; Spark/Rialto DH population located between 20.1 and 73.8 Mb, Getie et al., 2016; GWAS study, located between 68.9 and 71.0 Mb, Prins et al., 2016; LMPG-6/PI 362698-1 population located between 62.2 and 72.9 Mb, Zurn et al., 2018) and is likely the gene *Sr12*.

The *Sr12* gene (*QSr.cdl-3B.2*) alone did not provide strong adult plant resistance in three environments, suggesting the necessity of combining *Sr12* and other QTL(s). The gene *Lr34/Sr57* has been reported to enhance the effectiveness of Thatcher resistance in North America and Kenya (Kolmer et al., 2011), but the parents (LMPG-6 and CI 14275) were both negative for the *Lr34* gene-based marker (CsLV34), indicating that the resistance in CI 14275 did not involve *Lr34*/*Sr57*. Lack of significant QTL on chromosome 7D in this study, where *Lr34* is located, also is a strong indication that *Lr34*/*Sr57* was not segregating in this population. The KASP markers linked to *QSr.cdl-3B.2* were not associated with stem rust response in the validation population. To look into this further, we genotyped CI 14275, LMPG-6, line #162, and Kwale with the *Sr12*-associated marker from Hiebert et al. (2016): NB-LRR3. We found that LMPG-6 did not possess the *Sr12* gene, but CI 14275 and both parents of the validation population (Line #162 and Kwale) did possess *Sr12*. Therefore, *Sr12* is likely fixed in the validation population.

*QSr.cdl-4AL*, which explained a large proportion of the phenotypic variance (42.3%) for response to race TTTTF, is located at 73.3 Mb on chromosome arm 4AL. *QSr.cdl-4AL* is flanked by the markers Tdurum_contig42019_1714 and BS00009680_51. *SrND643*, a temporarily designated gene that is also located on chromosome arm 4AL, is reported to provide inadequate resistance under high disease pressure (Basnet et al., 2015). Even though *SrND643* is located between 72.3 and 73.7 Mb at a similar location to identified *QSr.cdl-4AL*, *SrND643* shows a low infection type of 2 to 22+ at the seedling stage against Ug99 races TTKSK and TTKST (Basnet et al., 2015). In contrast, line CI 14275 shows a high infection type of 3 to 33+ against Ug99 race TTKSK. These data suggest that *QSr.cdl-4AL* is not *SrND643*. Notably, CI 14275 and *SrND643* were tested against the same isolate under the same temperature regime and in the same greenhouse but not in the same experiment. The stem rust resistance gene *Sr7* with two characterized alleles *Sr7a* and *Sr7b* is located on chromosome arm 4AL, and these alleles confer resistance to some North American *Pgt* races (McIntosh et al., 1995; Turner et al., 2016). In this study, race TTTTF was virulent on *Sr7b* in the North American stem rust differential set with infection types ranging from 3− to 3+, ruling out the possibility of *Sr7b* being involved in the observed resistance. The QTL *QSr.rwg-4A* (believed to be *Sr7a*), is located between 59.1 and 73.9 Mb and expresses resistance to race TTTTF (Saini et al., 2018) in the durum wheat ‘Lebsock.’ The location of *QSr.cdl-4AL* and *QSr.rwg-4A* overlaps, and it is, therefore, likely that *QSr.cdl-4AL* identified in this study is *Sr7a*. *Sr7a* is demonstrated to be effective to race TTTTF by Turner et al. (2016). The detection of *QSr.cdl-4AL* in ETH18 may have been the result of an abundance of race TKTTF present in the disease nursery. Race TKTTF became predominant in Ethiopia starting in 2014 and was reported to be avirulent to *Sr7a* (Olivera et al., 2015).

*QSr.cdl-6A*, located between 51.4 and 52.0 Mb, conferred adult plant resistance in the ETH16, ETH18, STP16, and STP17 environments. *QSr.cdl-6A* explains phenotypic variance ranging from 6.4 to 12.6% and is flanked by the markers BS00023627_51, IAAV3806, and Excalibur_c60006_452 in various environments. The previously described stem rust resistance genes on chromosome arm 6AL are *Sr13*, *Sr26*, and *Sr52*. All three of these genes are derived from species other than bread wheat and confer seedling resistance to race TTKSK (Qi et al., 2011; Zhang et al., 2017). Because CI 14275 is not effective to race TTKSK at the seedling stage, these genes are not present in CI 14275. *QSr.cdl-6A* is close to both the QTL reported by Letta et al. (2013), which mapped between 60.6 and 61.8 Mb on 6A, and QTL reported in a genome-wide association study located between 10.0 and 59.6 Mb on 6A (Kankwatsa et al., 2017). The susceptibility of CI 14275 at the seedling stage to race TTKSK and pedigree information, however, rule out the possibilities of these genes (*Sr13*, *Sr26*, and *Sr52*) contributing to the observed resistance. The *Sr8* locus alleles *Sr8a* and *Sr8b* are reported on chromosome arm 6AS (Singh and McIntosh, 1986). A QTL identified by Bajgain et al. (2015) in an association mapping study is located at 52.3 Mb, a similar position to *QSr.cdl-6A*. Prins et al. (2016) identified QTL on 6AS although the physical location of linked markers was not found. A race-specific gene on 6A, likely *Sr8a*, was identified at the seedling stage for race TRTTF (Dunckel et al., 2015). In Ethiopian nurseries, other than the predominance of race TTKSK, *Pgt* races, including TRTTF and JRCQC, have been reported (Olivera et al., 2012; Hailu et al., 2015). The races TRTTF and JRCQC are avirulent to *Sr8a* although race TTKSK, which is predominant in Kenyan and Ethiopian environments, is virulent to *Sr8a* (Olivera et al., 2012; Hailu et al., 2015). The susceptibility of the RIL population to TRTTF rules out the possibility of *QSr.cdl-6A* being *Sr8a*. Nirmala et al. (2017) mapped *Sr8155B1*, a gene conferring resistance to race TTKST on chromosome 6A. *Sr8155B1* is located between 6.7 and 10.9 Mb, a different location from the QTL *QSr.cdl-6A* identified in this study. In addition, data from our laboratory indicate that TRTTF is avirulent to *Sr8155B1*, ruling out the possibility of CI 14275 possessing *Sr8155B1* because of its susceptibility to TRTTF (Table 1). The KASP markers linked to *QSr.cdl-6A* are not associated with stem rust resistance in the validation population. Unfortunately, line #162 (the resistant parent in the validation population) possesses the susceptible alleles of the *QSr.cdl-6A-*linked markers. Selecting line #162 as a parent was undertaken based on the phenotypic data alone. Therefore, the absence of *QSr.cdl-6A* from line #162 precludes our ability to validate this QTL in the validation population. Additional experiments are needed to validate the effectiveness of *QSr.cdl-6A*.

## Conclusion

This study reports QTL for wheat stem rust resistance on chromosomes 2BS, 3B, 4AL, and 6A. The identified QTL *QSr.cdl-2BS.2*, which conferred adult plant resistance in the KEN16, ETH16, and ETH18 environments, was validated in a second population in KEN18 and can be selected for by the validated linked marker Excalibur_c7963_1722. This QTL, therefore, has the potential of being used in marker-assisted selection. Our study is the first to both detect and validate an adult plant stem rust resistance QTL on chromosome arm 2BS. *QSr.cdl-3B.2*, which confers resistance in the KEN16, KEN17, KEN18, STP17, and STP18 environments is likely *Sr12*. *QSr.cdl-4AL*, which confers resistance to race TTTTF, is postulated to be *Sr7a*. *QSr.cdl-6A*, which confers resistance in the ETH16, ETH18, STP16, and STP17 environments, is potentially a new QTL, but the QTL requires validation in another population before it is recommended for use in breeding. The adult plant resistance of wheat line CI 14275 in Africa is characterized as the cumulative contribution of three QTL: *QSr.cdl-2BS.2*, *QSr.cdl-3B.2*, and *QSr.cdl-6A*.

## Data Availability Statement

The datasets generated for this study can be found in online repositories. The names of the repository/repositories and accession number(s) can be found in the article/Supplementary Material.

## Author Contributions

ZK, RD-M, and MNR contributed to writing of the initial drafts of the manuscript and provided insights on data analysis. YJ developed the mapping population and contributed to manuscript writing. EE helped with genotypic data analysis. WB, AG, GM, MSR, and SB facilitated the field studies in Africa. All authors contributed to the submitted manuscript.

## Conflict of Interest

The authors declare that the research was conducted in the absence of any commercial or financial relationships that could be construed as a potential conflict of interest.

## Supplementary Material

Supplementary Table 1 Genetic map of the LMPG-6/CI14275 population.

Supplementary Table 2 Seedling infection types of 113 recombinant inbred lines (RILs) of the cross LMPG-6/CI 14275 and the parents against five *Puccinia graminis* f. sp. *tritici* races: RTQQC, TTTTF, TPMKC, TRTTF, and TTKSK.

Supplementary Table 3 Stem rust severity and response of 113 recombinant inbred lines (RILs) of the cross LMPG-6/CI 14275 and the parents to predominant Ug99 races in Kenya in 2016 (KEN16), 2017 (KEN17), and 2018 (KEN18). Data for two replicates (Rep) are displayed.

Supplementary Table 4 Stem rust severity and response of 113 recombinant inbred lines (RILs) of the cross LMPG-6/C I14275 and the parents to predominant Ug99 races in Ethiopia in 2016 (ETH16), 2017 (ETH17), and 2018 (ETH18). Data for two replicates (Rep) are displayed.

Supplementary Table 5 Stem rust severity and response of 113 recombinant inbred lines (RILs) of the cross LMPG-6/C I14275 and the parents to US *Pgt* races in St. Paul, MN, in 2017 (STP17) and 2018 (STP18). Data for the two replicates are displayed.
