# Supplementary material for: Mapping and Validation of Stem Rust Resistance Loci in Spring Wheat Line CI 14275
Source: Front Plant Sci. 2021 Jan 12;11:609659. doi: 10.3389/fpls.2020.609659 (PMC7835402; doi:10.3389/fpls.2020.609659)
Supplement: Supplementary Table 2 — Seedling infection types of 113 recombinant inbred lines (RILs) of the cross LMPG-6/CI 14275 and the parents against five Puccinia graminis f. sp. tritici races: RTQQC, TTTTF, TPMKC, TRTTF, and TTKSK. [file Table_2.DOCX]

**Supplementary Table 2.** Seedling infection types of 113 recombinant inbred lines (RILs) of the cross LMPG-6/CI 14275 and the parents against five *Puccinia graminis* f. sp. *tritici* races RTQQC, TTTTF, TPMKC, TRTTF and TTKSK^a^

|  | RTQQC | |  | TTTTF | |  | TPMKC | |  | TRTTF | |  | TTKSK | |
| --- | --- | --- | --- | --- | --- | --- | --- | --- | --- | --- | --- | --- | --- | --- |
| Line Number /Name | Rep I | Rep II |  | Rep I | Rep II |  | Rep I | Rep II |  | Rep I | Rep II |  | Rep I | Rep II |
| 1 | 3+ (9) | 3+ (9) |  | 3+ (9) | 3+ (9) |  | 3+2- (7) | 3+ (9) |  | 33+ (8) | 33+ (8) |  | 33+ (8) | 3 (8) |
| 2 | 33+ (8) | 3+ (9) |  | 1+3+C (5) | ;13C (3) |  | 2+3+ (7) | 23- (6) |  | 3+ (9) | 3+ (9) |  | 3+3 (9) | 3+ (9) |
| 3 | 3+ (9) | 3+ (9) |  | 3+2- (7) | 3+ (9) |  | 3+3- (8) | 3+2+ (8) |  | 3 (8) | 3+ (9) |  | 33+ (8) | 3+ (9) |
| 4 | 33+ (8) | 33+ (8) |  | 3+3- (8) | 3+ (9) |  | 3+3- (8) | 33- (8) |  | 3 (8) | 3+ (9) |  | 3+ (9) | 3+ (9) |
| 5 | 3+ (9) | 23+ (6) |  | 1+3C (5) | 13- (4) |  | 2+3+ (7) | 23- (6) |  | 33- (8) | 3-C (7) |  | 3+ (9) | 3+ (9) |
| 6 | 3 (8) | 23+ (6) |  | 3+ (9) | 3 (8) |  | 2+3- (6) | 23- (6) |  | 33+ (8) | 33+ (8) |  | 33+ (8) | 3+ (9) |
| 7 | 3 (8) | 3+ (9) |  | 3- (7) | ;1 (1) |  | 2+3+ (7) | 23- (6) |  | 3 (8) | 33+ (8) |  | 3 (8) | 3+ (9) |
| 8 | 3+ (9) | 3+ (9) |  | 1+3C (5) | 33C (8) |  | 3+3 (9) | 3+3- (8) |  | 33+ (8) | 3+ (9) |  | 3+ (9) | 3+ (9) |
| 9 | 3+ (9) | 33+ (8) |  | 3+3C (9) | 3+ (9) |  | 23+ (6) | 23 (6) |  | 33- (8) | 3+ (9) |  | 3+ (9) | 3+ (9) |
| 10 | 3 (8) | 33+ (8) |  | 3+3- (8) | 3+ (9) |  | 23- (6) | 23 (6) |  | 33+ (8) | 33+ (8) |  | 3 (8) | 3+ (9) |
| 11 | 3+ (9) | 3+3 (9) |  | 3+3- (8) | 3+3- (8) |  | 23+ (6) | 3+3 (9) |  | 3- (7) | 3+ (9) |  | 3 (8) | 3+ (9) |
| 12 | 3+ (9) | 3+ (9) |  | 0, 1+ (0) | 13 (4) |  | 3+ (9) | 3+3 (9) |  | 33C (8) | 3-C (7) |  | 3 (8) | 3+ (9) |
| 13 | 3+ (9) | 33+ (8) |  | 3+ (9) | 3+ (9) |  | 3+3 (9) | 3+ (9) |  | 3 (8) | 3+ (9) |  | 33+ (8) | 3+ (9) |
| 14 | 33+ (8) | 33+ (8) |  | 3+3- (8) | 3+3- (8) |  | 23- (6) | 23- (6) |  | 3 (8) | 33+ (8) |  | 33+ (8) | 33+ (8) |
| 15 | 3+ (9) | 3+ (9) |  | 3+ (9) | 3+ (9) |  | 32- (7) | 33- (8) |  | 3 (8) | 33+ (8) |  | 33+ (8) | 3+ (9) |
| 16 | 3+ (9) | 3+ (9) |  | 3+ (9) | 3+ (9) |  | 3+3- (8) | 3+3 (9) |  | 33+ (8) | 3+ (9) |  | 33+ (8) | 3+ (9) |
| 17 | 3+ (9) | 3+ (9) |  | 3+3- (8) | 3+3- (8) |  | 3+3- (8) | 3+ (9) |  | 33- (8) | 3+3- (8) |  | 3+ (9) | 3+ (9) |
| 18 | 3+ (9) | 3+ (9) |  | 3+ (9) | 3+ (9) |  | 33+ (8) | 3+3 (9) |  | 3- (7) | 33- (8) |  | 3+ (9) | 3+ (9) |
| 19 | 3+ (9) | 3+ (9) |  | 3+3- (8) | 3+ (9) |  | 3+ (9) | 3+3 (9) |  | 3- (7) | 3- (7) |  | 33+ (8) | 3+ (9) |
| 20 | 3+ (9) | 33+ (8) |  | 0/1+3- (0/4) | 22+ (5) |  | 3+3- (8) | 33- (8) |  | 33+ (8) | 33+C (8) |  | 3 (8) | NA |
| 21 | 3+ (9) | 3+ (9) |  | 3+3- (8) | 3+ (9) |  | 3+3 (9) | 3+ (9) |  | 33+ (8) | 3+ (9) |  | 33+ (8) | 3+ (9) |
| 22 | 3+3 (9) | 33+ (8) |  | 0/1+ (0/3) | 33- (8) |  | 33- (8) | 23 (6) |  | 33+ (8) | 33+ (8) |  | 3+ (9) | 3+ (9) |
| 23 | 3+3 (9) | 33+ (8) |  | 3+3- (8) | 3+ (9) |  | 23- (6) | 23- (6) |  | 33+ (8) | 3+ (9) |  | 3+ (9) | 3+ (9) |
| 24 | 33+ (8) | 33+ (8) |  | 3+3- (8) | 3+3- (8) |  | 23- (6) | 3-3+ (8) |  | 33+ (8) | 33+ (8) |  | 33+ (8) | 3+ (9) |
| 25 | 3+ (9) | 3+ (9) |  | 3+ (9) | 3+ (9) |  | 3+ (9) | 3+3- (8) |  | 3+ (9) | 3+ (9) |  | 3+ (9) | 3+ (9) |
| 26 | 33- (8) | 3+ (9) |  | 3+3- (8) | - |  | 3+ (9) | 3 (8) |  | - |  |  | 33- (8) | 33-C (8) |
| 27 | 32+ (7) | 33+ (8) |  | 1+3+ (5) | ;1 (1) |  | 3+3- (8) | 3+ (9) |  | 3+ (9) | 33+ (8) |  | 33+ (8) | 3C (8) |
| 28 | 32- (7) | 33+ (8) |  | 3+1+C (7) | 3C (8) |  | 3+3- (8) | 3+ (9) |  | 33+ (8) | 3+ (9) |  | 33- (8) | 3+ (9) |
| 29 | 23- (6) | 3- (7) |  | 3+ (9) | 3+ (9) |  | 3+ (9) | 23 (6) |  | 33+ (8) | 3+ (9) |  | 3 (8) | 3+ (9) |
| 30 | 3+2- (7) | 3+ (9) |  | 3+1/0 (7) | ;13 (3) |  | 3+3- (8) | 3+3- (8) |  | 33+(8) | 33+ (8) |  | 3+3 (9) | 3+ (9) |
| 31 | 23- (6) | 33+ (8) |  | 33+C (8) | 3+ (9) |  | 3+3- (8) | 3+3- (8) |  | 33+ (8) | 3+ (9) |  | 3+ (9) | 3+ (9) |
| 32 | 23- (6) | 33+ (8) |  | 3+ (9) | 13 (4) |  | 2+3+ (7) | 32+ (7) |  | 33+ (8) | 3+ (9) |  | 3+ (9) | 3+ (9) |
| 33 | 3 (8) | 3+ (9) |  | 3+ (9) | ;13 (3) |  | 3+2 (8) | 3+3- (8) |  | 33+ (8) | 3+3 (9) |  | 3+ (9) | 3 (8) |
| 34 | 2+ (6) | 3+ (9) |  | 1+3C (5) | 1 (2) |  | 3+2 (8) | 3- (7) |  | 33+ (8) | 33+ (8) |  | 3+ (9) | 3+ (9) |
| 35 | 22+ (5) | 3- (7) |  | 1+3+C (5) | 1 (2) |  | 23+ (6) | 23+ (6) |  | 33+ (8) | 3+C (9) |  | 3+ (9) | 3+ (9) |
| 36 | 2+ (6) | 3 (8) |  | 13C (4) | 13- (4) |  | 2+3 (7) | 23 (6) |  | 33+ (8) | 3+ (9) |  | 3 (8) | 3+ (9) |
| 37 | 22+ (5) | 3 (8) |  | 13 (4) | ;1 (1) |  | 23+ (6) | 32 (7) |  | 33+ (8) | 33+ (8) |  | 3+ (9) | 3+ (9) |
| 38 | 22+ (5) | 3- (7) |  | 31C (6) | 13- (4) |  | 23- (6) | 23- (6) |  | 33C (8) | 3-C (7) |  | 3+ (9) | 3+ (9) |
| 39 | 3+ (9) | 3+ (9) |  | 1+3C (5) | 1 (2) |  | 3+3- (8) | 32- (7) |  | 33+ (8) | 33+ (8) |  | 3+ (9) | 3+ (9) |
| 40 | 2+3- (6) | 3+ (9) |  | 1+3+C (5) | 33C (8) |  | 3+3- (8) | 23 (6) |  | 33+ (8) | 3+3 (9) |  | 3+ (9) | 3+ (9) |
| 41 | 1+ (3) | 23- (6) |  | 1+3+C (5) | 31C (6) |  | 23+ (6) | 22+ (5) |  | 33+ (8) | 33+ (8) |  | 3+ (9) | 3+ (9) |
| 42 | 33+ (8) | 3+ (9) |  | 3+ (9) | 3+ (9) |  | 33+ (8) | 3+2 (8) |  | 33+ (8) | 33+ (8) |  | 3+ (9) | 3+ (9) |
| 43 | 22+ (5) | 3+ (9) |  | 3+ (9) | 32 (7) |  | 33- (8) | 32 (7) |  | 33+ (8) | 3+ (9) |  | 3+ (9) | 3+ (9) |
| 44 | 23- (6) | 3+ (9) |  | 3+1C (7) | 13C (4) |  | 33+ (8) | 3+2 (8) |  | 3+3 (9) | 3+ (9) |  | 3+ (9) | 3+ (9) |
| 45 | 33+ (8) | 3+ (9) |  | 3+C (9) | ;1 (1) |  | 3+3- (8) | 33+ (8) |  | 3-C (7) | 33+C (8) |  | 3+ (9) | 3+ (9) |
| 46 | 23- (6) | 3+ (9) |  | 13+C (4) | 31C (6) |  | 3+ (9) | 32+ (7) |  | 3- (7) | 3 (8) |  | 3+ (9) | 3+ (9) |
| 47 | 22+ (5) | 3+ (9) |  | 1+3C (5) | ;1 (1) |  | 23 (6) | 3+2 (8) |  | 3-C (7) | 3-C (7) |  | 3+ (9) | 3+ (9) |
| 48 | 3+ (9) | 3+3- (8) |  | 1+3-C (4) | ;1 (1) |  | 23+ (6) | 22+ (5) |  | 33+ (8) | 33+C (8) |  | 33+ (8) | 3+ (9) |
| 49 | 3+ (9) | 3+ (9) |  | 0 (0) | 1 (2) |  | 3+3- (8) | 3+2 (8) |  | 3+3C (9) | 3- (7) |  | 3+ (9) | 3+ (9) |
| 50 | 3+ (9) | 3+ (9) |  | ; 13- (2) | ;1 (1) |  | 3+ (9) | 3+3- (8) |  | 3+3 (9) | 3+ (9) |  | 3 (8) | 3+ (9) |
| 51 | 33+ (8) | 3- (7) |  | 3+3C (9) | 31C (6) |  | 3+2 (8) | 22+ (5) |  | 3+ (9) | 3+ (9) |  | 33- (8) | 3+ (9) |
| 52 | 3+ (9) | 3+ (9) |  | 1+3C (5) | 13- (4) |  | 3+2 (8) | 33+ (8) |  | 3+ (9) | 3+3C (9) |  | 33+ (8) | 3+ (9) |
| 53 | 33+ (8) | 3- (7) |  | 1+3-C (4) | ;13- (2) |  | 23- (6) | 2+ (6) |  | 33+ (8) | 33+ (8) |  | 3+ (9) | 3+ (9) |
| 54 | 3+ (9) | 3+ (9) |  | 3+ (9) | 3+ (9) |  | 3+ (9) | 3+3- (8) |  | 3+ (9) | 3+ (9) |  | 3+ (9) | 3+ (9) |
| 55 | 3+ (9) | 3+ (9) |  | 1+3+ (5) | ;1+ (1) |  | 3+ (9) | 33+ (8) |  | 3+3 (9) | 3+ (9) |  | 3+3 (9) | 3+ (9) |
| 56 | 3+ (9) | 3 (8) |  | 3+ (9) | 3+ (9) |  | 23+ (6) | 23+ (6) |  | 3+ (9) | 3+ (9) |  | 33- (8) | 3+ (9) |
| 57 | 3+ (9) | 3+ (9) |  | 1+3- (4) | 31C (6) |  | 3+2 (8) | 32- (7) |  | 3-C (7) | 3+ (9) |  | 33+ (8) | 3+ (9) |
| 58 | 3+ (9) | 3+ (9) |  | 3+3C (9) | 3+ (9) |  | 3+3- (8) | 32 (7) |  | 33+ (8) | 3+ (9) |  | 3 (8) | 3+ (9) |
| 59 | 3+ (9) | 3+ (9) |  | 1+3C (5) | 31C (6) |  | 3+3- (8) | 3+3 (9) |  | 33+ (8) | 3+(9) |  | 33-C (8) | 3+ (9) |
| 60 | 3- (7) | 23- (6) |  | 0/1+ (0/3) | ;1 (1) |  | 23- (6) | 22+ (5) |  | 3-C (7) | 3-C (7) |  | 3- (7) | 33- (8) |
| 61 | 3- (7) | 23- (6) |  | 1+3+C (5) | 32- (7) |  | 32 (7) | 2+2 (6) |  | 3 (8) | 33+ (8) |  | 33- (8) | 3 (8) |
| 62 | 3+3 (9) | 3+ (9) |  | 33+ (8) | 3+ (9) |  | 3+ (9) | 32+ (7) |  | 33+ (8) | 3+ (9) |  | 3 (8) | 3+ (9) |
| 63 | 3+ (9) | 3+ (9) |  | 3+3- (8) | 3+ (9) |  | 3+ (9) | 33+ (8) |  | 33- (8) | 3+ (9) |  | 3 (8) | 33+ (8) |
| 64 | 3+ (9) | 3+c (9) |  | 1+3+C (5) | 31C (6) |  | 3+ (9) | 33+ (8) |  | 3- (7) | 3+ (9) |  | 3+ (9) | 3+ (9) |
| 65 | 3+ (9) | 33+ (8) |  | 1+3C (5) | 31C (6) |  | 33+ (8) | 23+ (6) |  | 33- (8) | 33+C (8) |  | 33+ (8) | 3+ (9) |
| 66 | 3+3- (8) | 3- (7) |  | 0/1+C (0/3) | 13 (4) |  | 3+3- (8) | 3+3 (9) |  | 3-C (7) | 3-C (7) |  | 33+ (8) | 3+ (9) |
| 67 | 33+ (8) | 13- (4) |  | 3 (8) | 3+ (9) |  | 3+3- (8) | 3+ (9) |  | 33+ (8) | 3+ (9) |  | 3 (8) | 3+ (9) |
| 68 | 3+ (9) | 3+ (9) |  | 1+3C (5) | 31C (6) |  | 3+ (9) | 33+ (8) |  | 33- (8) | 3-C (7) |  | 3+ (9) | 3+ (9) |
| 69 | 3+ (9) | 3-3+ (8) |  | 1+3- (4) | 13C (4) |  | 3+3 (9) | 3+3 (9) |  | 3-3+ (8) | 3-3+ (8) |  | 3+ (9) | 3+ (9) |
| 70 | 3+ (9) | 13- (4) |  | ;1 (1) | 13C (4) |  | 33+ (8) | 33+ (8) |  | 33+C (8) | 33+C (8) |  | 3+ (9) | 3+ (9) |
| 71 | 33- (8) | ;13- (2) |  | 13C (4) | 31C (6) |  | 23- (6) | 23- (6) |  | 3- (7) | 3C (8) |  | 3 (8) | 33+ (8) |
| 72 | 33- (8) | ;13- (2) |  | 3+1 (7) | 3+ (9) |  | 3- (7) | 32+ (7) |  | 3 (8) | 33+ (8) |  | 3+ (9) | 3+ (9) |
| 73 | 3+ (9) | 3+ (9) |  | 3+3- (8) | 3+ (9) |  | 33+ (8) | 33+ (8) |  | 33+ (8) | 33+ (8) |  | 3 (8) | 3- (7) |
| 74 | 3+ (9) | 3+ (9) |  | 33+ (8) | 33C (8) |  | 3+3- (8) | 23- (6) |  | 33+ (8) | 33+ (8) |  | 3+ (9) | 3+ (9) |
| 75 | 3+3- (8) | 33+ (8) |  | 3+ (9) | 3+ (9) |  | 3+ (9) | 3+3 (9) |  | 3+ (9) | 3+ (9) |  | 3+ (9) | 3+ (9) |
| 76 | 33+ (8) | 3+ (9) |  | 33+ (8) | 13C (4) |  | 23+ (6) | 23+ (6) |  | 3+ (9) | 3C (8) |  | 3 (8) | 3+ (9) |
| 77 | 3+ (9) | 3 (8) |  | 13C (4) | 31C (6) |  | 3+ (9) | 23- (6) |  | 33+ (8) | 33+ (8) |  | 3+ (9) | 3+ (9) |
| 78 | 3+3 (9) | 33+ (8) |  | 1+3C (5) | 13C (4) |  | 3+3- (8) | 32 (7) |  | 3+ (9) | 3C (8) |  | 3+3 (9) | 3+3 (9) |
| 79 | 3+3- (8) | 32- (7) |  | 13-C (4) | 13 (4) |  | 3+ (9) | 3- (7) |  | 3-C (7) | 3C (8) |  | 3+ (9) | 3+3 (9) |
| 80 | 3+ (9) | 2+ (6) |  | 3+ (9) | 3+ (9) |  | 3-2+ (7) | 2/3+ (6) |  | 3+ (9) | 31 (6) |  | 3+ (9) | 3+ (9) |
| 81 | 33- (8) | 3 (8) |  | 3+3-C (8) | 3+ (9) |  | 3+ (9) | 23- (6) |  | 33+ (8) | 3-C (7) |  | 3+ (9) | 3+ (9) |
| 82 | 3 (8) | 3+ (9) |  | 3+ (9) | 3+ (9) |  | 3+ (9) | 23+ (6) |  | 3+ (9) | 33+ (8) |  | 3+ (9) | 3+ (9) |
| 83 | 3+3- (8) | 3 (8) |  | 13- (4) | 13C (4) |  | 3+2- (7) | 23- (6) |  | 3+ (9) | 3C (8) |  | 3+ (9) | 3+ (9) |
| 84 | 3+ (9) | 33+ (8) |  | 33+ (8) | 3 (8) |  | 33+ (8) | 23- (6) |  | 3+ (9) | 33+ (8) |  | 3+ (9) | 3+ (9) |
| 85 | 3+3- (8) | 33- (8) |  | 3+1C (7) | 3+ (9) |  | 3+3- (8) | 23+ (6) |  | 3+ (9) | 3+ (9) |  | 3+ (9) | 3+ (9) |
| 86 | 3+ (9) | 3+ (9) |  | 3+ (9) | 3+ (9) |  | 3+ (9) | 3+3 (9) |  | 3+ (9) | 3+ (9) |  | 3+ (9) | 3+ (9) |
| 87 | 3+ (9) | 3+ (9) |  | 13C (4) | 3+ (9) |  | 33+ (8) | 3+3 (9) |  | 3+3- (8) | 33- (8) |  | 3+ (9) | 3+ (9) |
| 88 | 3+ (9) | 3+ (9) |  | 3+ (9) | 3+ (9) |  | 3+ (9) | 33+ (8) |  | 3+3- (8) | 33+ (8) |  | 3+ (9) | 3+ (9) |
| 89 | 3+3- (8) | 33+ (8) |  | 13+ (4) | ;1 (1) |  | 23+ (6) | 23- (6) |  | 33- (8) | 3- (7) |  | 3+ (9) | 3+ (9) |
| 90 | 3+ (9) | 3+ (9) |  | 3+ (9) | 3+3 (9) |  | 3+ (9) | 33- (8) |  | 33+ (8) | 33+ (8) |  | 3+ (9) | 3+ (9) |
| 91 | 3 (8) | 13- (4) |  | 13- (4) | 13C (4) |  | 33+ (8) | 23- (6) |  | 3+3C (9) | 3-C (7) |  | 3+ (9) | 3+ (9) |
| 92 | 3+ (9) | 3 (8) |  | 1+3C (5) | 31C (6) |  | 23+ (6) | 32 (7) |  | 3-C (7) | 33C (8) |  | 3+ (9) | 3+ (9) |
| 93 | 3+ (9) | 3+ (9) |  | 3+ (9) | 3+ (9) |  | 3+ (9) | 32+ (7) |  | 3C (8) | 33+ (8) |  | 3+ (9) | 3+ (9) |
| 94 | 3+3- (8) | 33+ (8) |  | 3+ (9) | 3+ (9) |  | 3+ (9) | 3+3 (9) |  | 3+ (9) | 33+ (8) |  | 3+ (9) | 3+ (9) |
| 95 | 3+ (9) | 3+ (9) |  | 3+3C (9) | ;1 (1) |  | 3+ (9) | 3+ (9) |  | 3+ (9) | 33+ (8) |  | 3+ (9) | 3+ (9) |
| 96 | 33+ (8) | 3- (7) |  | 13C (4) | 13C (4) |  | 23+ (6) | 23- (6) |  | 3C (8) | 33+C (8) |  | 3+ (9) | 3+ (9) |
| 97 | 33- (8) | 3- (7) |  | 31 (6) | 13 (4) |  | 3+ (9) | 23+ (6) |  | 3- (7) | 3-C (7) |  | 3+ (9) | 3+ (9) |
| 98 | 3+ (9) | 3- (7) |  | 33+C (8) | 13 (4) |  | 3+ (9) | 23- (6) |  | 3- (7) | 33+ (8) |  | 3+ (9) | 33+ (8) |
| 99 | 3+ (9) | 3+ (9) |  | 3+ (9) | ;1 (1) |  | 3+ (9) | 3+ (9) |  | 33+ (8) | 33+ (8) |  | 3+ (9) | 3+ (9) |
| 100 | 3+ (9) | 3+ (9) |  | 1+3C (5) | 13 (4) |  | 3+ (9) | 3+2 (8) |  | 3-C (7) | 33+ (8) |  | 3+ (9) | 3+ (9) |
| 101 | 3 (8) | 3+ (9) |  | 1+3+ (5) | 31C (6) |  | 3+2 (8) | 3-3+ (8) |  | 3- (7) | 3- (7) |  | 3+ (9) | 3+ (9) |
| 102 | 3+ (9) | 3+ (9) |  | 1+3 (5) | 33C (8) |  | 3+ (9) | 3+ (9) |  | 3- (7) | 3 (8) |  | 3+ (9) | 3+ (9) |
| 103 | 3+3- (8) | 3+ (9) |  | 3- (7) | 13 (4) |  | 3+2- (7) | 32 (7) |  | 3-C (7) | 33+ (8) |  | 33+ (8) | 3+ (9) |
| 104 | 3+ (9) | 3+ (9) |  | 3+ (9) | 3+ (9) |  | 3+ (9) | 32- (7) |  | 3+ (9) | 3+ (9) |  | 3+ (9) | 3+ (9) |
| 105 | 3+ (9) | 3+ (9) |  | 33+ (8) | 3+3 (9) |  | 3+3- (8) | 32 (7) |  | 33+ (8) | 33+ (8) |  | 3+ (9) | 3+ (9) |
| 106 | 3 (8) | 3- (7) |  | 33+ (8) | 31 (6) |  | 3+ (9) | 23- (6) |  | 3- (7) | 3+ (9) |  | 3+ (9) | 3+ (9) |
| 107 | 33+ (8) | 1+3C (5) |  | 33+C (8) | 31C (6) |  | 3+3- (8) | 23- (6) |  | 3-3+ (8) | 3+ (9) |  | 3+ (9) | 3+ (9) |
| 108 | 3+ (9) | 3+ (9) |  | 31 (6) | 31C (6) |  | 3+3- (8) | 3+2 (8) |  | 33+ (8) | 3+ (9) |  | - | 3+ (9) |
| 109 | 3+3- (8) | 3 (8) |  | 1+3- (4) | 31C (6) |  | 3+ (9) | 3+ (9) |  | 3- (7) | 33- (8) |  | 3+ (9) | 3 (8) |
| 110 | 3+ (9) | 3+ (9) |  | 3+ (9) | 3+ (9) |  | 3+ (9) | 3+2 (8) |  | 3+ (9) | 3+ (9) |  | 3+ (9) | 3+ (9) |
| 111 | 3+ (9) | 1+3C (5) |  | 1+3+C (5) | 13 (4) |  | 23- (6) | 32 (7) |  | 3- (7) | 33- (8) |  | 3+ (9) | 3 (8) |
| 112 | 33+ (8) | 3- (7) |  | 1+3- (4) | ;1+ (1) |  | 2+3- (6) | 32 (7) |  | 33+ (8) | 33+ (8) |  | 3+ (9) | 3+ (9) |
| 113 | 3+3- (8) | 3+ (9) |  | 1+3- (4) | 13C (4) |  | 3+ (9) | 3+3- (8) |  | 33+ (8) | 3-3+ (8) |  | 3+ (9) | 3+ (9) |
| C 14275 | 33+ (8) | 3 (8) |  | 1+3C (5) | ;1 (1) |  | 23- (6) | 23+ (6) |  | 33+ (8) | 33+ (8) |  | 33+ (8) | 33+ (8) |
| LMPG-6 | 3+/3 (9) | 3+ (9) |  | 3+ (9) | 3+ (9) |  | 3+3 (9) | 3+ (9) |  | 3+ (9) | 33+ (8) |  | 33+ (8) | 3 (8) |

^a^Infection types were score based on the 0-4 scale developed by Stakman *et al.* (1962). A forward slash (/) symbol separates multiple infection types observed on different plants of the same line. The plus (+) and minus (-) symbols were used for the pustules that were relatively larger or smaller, respectively, than normal. Plants with ITs ranging from 0 to 2 were categorized as resistant, and those with 3-4 ITs were categorized as susceptible. The seedling infection types based on a 0 to 4 scale were then converted to a 0 to 9 linear scale (indicated in parentheses) according to Gao *et al.* (2019).
