# Supplementary material for: Mapping and Validation of Stem Rust Resistance Loci in Spring Wheat Line CI 14275
Source: Front Plant Sci. 2021 Jan 12;11:609659. doi: 10.3389/fpls.2020.609659 (PMC7835402; doi:10.3389/fpls.2020.609659)
Supplement: Supplementary Table 4 — Stem rust severity and response of 113 recombinant inbred lines (RILs) of the cross LMPG-6/C I14275 and the parents to predominant Ug99 races in Ethiopia in 2016 (ETH16), 2017 (ETH17), and 2018 (ETH18). Data for two replicates (Rep) are displayed. [file Table_4.DOCX]

**Supplementary Table 4.** Stem rust severity and response of 113 recombinant inbred lines (RILs) of the cross LMPG-6/C I14275 and the parents to predominant Ug99 races in Ethiopia in 2016 (ETH16), 2017 (ETH17) and in 2018 (ETH18). Data for two replicates (Rep) are displayed^a^.

|  | ETH16 | |  | ETH17 | |  | ETH18 | |
| --- | --- | --- | --- | --- | --- | --- | --- | --- |
| Line Number/Name | Rep I | REP II |  | Rep I | Rep II |  | Rep I | Rep II |
| 1 | - | 40SMS (36) |  | 50SMS (45) | 40S (40) |  | 60S (60) | 60S (60) |
| 2 | - | 5R (1) |  | 10MS (8) | 20MS (18) |  | 25MSMR (15) | 20MRMS (12) |
| 3 | 30SMS (27) | 15MSS (13.5) |  | 40SMS (36) | 40S (40) |  | 30S (30) | 50SMS (45) |
| 4 | 60S (60) | 60S (60) |  | 20MSS (18) | 40S (40) |  | 60S (60) | 70S (70) |
| 5 | - | 30MRMS (18) |  | 20MSS (18) | 30MSS (27) |  | 50MSMR (30) | 40MSS (36) |
| 6 | 5MRMS (3) | 15MSS (13.5) |  | 40SMS (36) | 40S (40) |  | 50MS (40) | 30MS (24) |
| 7 | - | - |  | 15MS (12) | 20S (20) |  | 40MSMR (24) | 40MSMR (24) |
| 8 | 30MSS (27) | 40S (40) |  | 20MS (16) | 30MS (24) |  | 60S (60) | 50S (50) |
| 9 | - | 30MRMS (18) |  | 20SMS (18) | 20MS (16) |  | 40SMS (36) | 40MSS (36) |
| 10 | 25MS (20) | 30MSMR (18) |  | 20MS (16) | 30MSS (27) |  | 40MSMR (24) | 30MSS (27) |
| 11 | 10MSS (9) | 20MS (16) |  | 50S (50) | 40S (40) |  | 40S (40) | 40S (40) |
| 12 | 15SMS (13.5) | 15MS (12) |  | 30MSS (27) | 30MSS (27) |  | 30MS (24) | 40SMS (36) |
| 13 | - | 25MSMR (15) |  | 40MSS (36) | 40S (40) |  | 40MS (32) | 50SMS (45) |
| 14 | 30MSMR (18) | 25MRMS (15) |  | 20MSS (18) | 10MS (8) |  | 50MSS (45) | 50SMS (45) |
| 15 | 70S (70) | 60S (60) |  | 40SMS (36) | 40S (40) |  | 40S (40) | 50S (50) |
| 16 | 60S (60) | 60S (60) |  | 20MS (16) | 10MS (8) |  | 70S (70) | 30MS (24) |
| 17 | 30MRMS (18) | 60S (60) |  | 50SMS (45) | 30MSS (27) |  | 45SMS (40.5) | 55S (55) |
| 18 | 30SMS (27) | 40MS (32) |  | 40SMS (36) | 40S (40) |  | 60S (60) | 60S (60) |
| 19 | 20MSS (18) | 20MSS (18) |  | 40MSS (36) | 40SMS (36) |  | 50S (50) | 60S (60) |
| 20 | TSMR (0.2) | - |  | 10MS (8) | 5MS (4) |  | 20MS (16) | - |
| 21 | 50S (50) | 70S (70) |  | 30MS (24) | 40S (40) |  | 60S (60) | 70S (70) |
| 22 | - | 15MRMS (9) |  | 20MSS (18) | 20SMS (18) |  | 30MSMR (18) | 40MSS (36) |
| 23 | - | - |  | 30MSS (27) | 15MS (12) |  | 20MSS (18) | 20SMS (18) |
| 24 | 20SMS (18) | 20MSS (18) |  | 40S (40) | 40MSS (36) |  | 50SMS (45) | 50SMS (45) |
| 25 | 70S (70) | 60S (60) |  | 30MSS (27) | 40S (40) |  | 70S (70) | 70S (70) |
| 26 | 30SMS (27) | 30S (30) |  | - | - |  | 50S (50) | 70S (70) |
| 27 | 15SMS (13.5) | 10MSS (9) |  | 10MS (8) | 10MS (8) |  | 30MSS (27) | 15MSS (13.5) |
| 28 | 10SMS (9) | 15MSS (13.5) |  | 20MSS (18) | 25MS (20) |  | 30SMS (27) | 20MS (16) |
| 29 | 15SMS (13.5) | 15MSS (13.5) |  | 40MSS (36) | 40MSS (36) |  | 50SMS (45) | 40SMS (36) |
| 30 | 80S (80) | 60S (60) |  | 20MS (16) | 30MS (24) |  | 50S (50) | 60S (60) |
| 31 | 60S (60) | 40S (40) |  | 40SMS (36) | 20MS (16) |  | 55s (55) | 50S (50) |
| 32 | 30MSMR (18) | 20MRMS (12) |  | 15MS (12) | 30MSS (27) |  | 50SMS (45) | 40MSS (36) |
| 33 | 25MRMS (15) | 35S (35) |  | 40MSS (36) | 40MSS (36) |  | 50S (50) | 60S (60) |
| 34 | 50S (50) | 70S (70) |  | 40SMS (36) | 40S (40) |  | 50S (50) | 40SMS (36) |
| 35 | - | 5MS (4) |  | 15MSS (13.5) | 5MS (4) |  | 10MS (8) | TMR (0.2) |
| 36 | - | - |  | 10MS (8) | 50S (50) |  | 50S (50) | 40SMS (36) |
| 37 | 15MSS (13.5) | 15MSS (13.5) |  | 30SMS (27) | 30MSS (27) |  | 30MSS (27) | 30SMS (27) |
| 38 | 5MRMS (3) | 20MRMS (12) |  | 40SMS (36) | 30SMS (27) |  | 30MSMR (18) | 40SMS (36) |
| 39 | - | - |  | 50MSS (45) | 40MSS (36) |  | 60SMS (54) | 40S (40) |
| 40 | 30SMS (27) | - |  | 20MSS (18) | 30SMS (27) |  | 60SMS (54) | 40S (40) |
| 41 | 40SMS (36) | 50SMS (45) |  | 5MS (4) | 5MS (4) |  | 40MRMS (24) | 40SMS (36) |
| 42 | 50S (50) | 70S (70) |  | 40MSS (36) | 50S (50) |  | 60S (60) | 50S (50) |
| 43 | 50S (50) | 40S (40) |  | 30MS (24) | 50S (50) |  | 50S (50) | 60S (60) |
| 44 | 25SMS (22.5) | - |  | 30MSS (27) | 30MSS (27) |  | 25MS (20) | 40SMS (36) |
| 45 | 10MRMS (6) | - |  | 20MSS (18) | 20MS (16) |  | 15MSS (13.5) | 20MS (16) |
| 46 | 20MSS (18) | 30MSS (27) |  | 15MSS (13.5) | 30MSS (27) |  | 30SMS (27) | 40SMS (36) |
| 47 | 40SMS (36) | 30MSMR (18) |  | 5MSS (4.5) | 10MS (8) |  | 60MSMR (36) | 30SMS (27) |
| 48 | 15MRMS (9) | 15MSS (13.5) |  | 20MS (16) | 40MSS (36) |  | 40MS (32) | 40MSMR (24) |
| 49 | 25MS (20) | 50MSMR (30) |  | 10MSS (9) | 40SMS (36) |  | 50SMS (45) | 30MSS (27) |
| 50 | 30MRMS (18) | 30MRMS (18) |  | 15MSS (13.5) | 20MS (16) |  | 40MSMR (24) | 30MSS (27) |
| 51 | 70S (70) | 60S (60) |  | 30MSS (27) | 30MS (24) |  | 60S (60) | 50MSS (45) |
| 52 | 20MSS (18) | 20SMS (18) |  | 30MSS (27) | 30MSS (27) |  | 50S (50) | 40SMS (36) |
| 53 | 5MR (2) | 15MR, 10S (6) |  | 15MS (12) | 20MS (16) |  | 25MSMR (15) | 30MSMR (18) |
| 54 | 25SMS (22.5) | 30SMS (27) |  | 50S (50) | 40MSS (36) |  | 40S (40) | 60S (60) |
| 55 | 20MSS (18) | 25SMS (22.5) |  | 10MS (8) | 30MSS (27) |  | 40SMS (36) | 15MRM (9) |
| 56 | 15MS (12) | 5MS (4) |  | 15MS (12) | 30S (30) |  | 50S (50) | 40MSS (36) |
| 57 | - | 30SMS (27) |  | 40MSS (36) | 40SMS (36) |  | 50S (50) | 60S (60) |
| 58 | 30MSS (27) | 30MSMR (18) |  | 30MSS (27) | 50S (50) |  | 50SMS (45) | 40SMS (36) |
| 59 | 30SMS (27) | 20SMS (18) |  | 25MS (20) | 25MS (20) |  | 50SMS (45) | 50MSMR (30) |
| 60 | - | - |  | 5MS (4) | 10MS (8) |  | 10MRR (3) | 10MSMR (6) |
| 61 | 20MSMS (16) | 30MRMS (18) |  | 20MS (16) | 20MSS (18) |  | - | 40MSS (36) |
| 62 | TR (0.2) | 5MS (4) |  | 2SMS (20) | 30MSS (27) |  | 30MS (24) | 20MSS (18) |
| 63 | 60S (60) | 50S (50) |  | 40MSS (36) | 30MSS (27) |  | 40S (40) | 60S (60) |
| 64 | 30SMS (27) | 15MSS (13.5) |  | 30S (30) | 20MS (16) |  | 60S (60) | 60S (60) |
| 65 | 5MS (4) | - |  | 20MS (16) | 20MS (16) |  | 5MS (4) | 40MSMR (24) |
| 66 | 10MSMR (6) | 15SMS (13.5) |  | 30MSS (27) | 40SMS (36) |  | 30SMS (27) | 20MSMR (12) |
| 67 | 5SMS (4.5) | 15SMS (13.5) |  | 40MSS (36) | 40S (40) |  | 40S (40) | 30SMS (27) |
| 68 | - | 50S (50) |  | 30MSS (27) | 30SMS (27) |  | 50MSS (45) | 60S (60) |
| 69 | 25MSMR (15) | 5SMS (4.5) |  | 15MSS (13.5) | 5MS (4) |  | 30MSS (27) | 10MSMR (6) |
| 70 | 60S (60) | 70S (70) |  | 15MSS (13.5) | 10MS (8) |  | 60MSS (54) | 60SMS (54) |
| 71 | 15SMS (13.5) | 30MR (12) |  | 30MSS (27) | 30MSS (27) |  | 40MRMS (24) | 25MSMR (15) |
| 72 | 40SMS (36) | 40MSMR (24) |  | 15MSS (13.5) | 15MS (12) |  | 60S (60) | 55S (55) |
| 73 | 15SMS (13.5) | 25SMS (22.5) |  | 30MSS (27) | 40SMS (36) |  | 50MSS (45) | 50SMS (45) |
| 74 | - | 30MSMR (18) |  | 30MSS (27) | 40MSS (36) |  | 50S (50) | 50S (50) |
| 75 | 10MS (8) | 15MSS (13.5) |  | 30MSS (27) | 40S (40) |  | 30MSS (27) | 30MSS (27) |
| 76 | 30SMS (27) | 25MR (10) |  | 10MS (8) | 10MS (8) |  | 50SMS (54) | 40S (40) |
| 77 | 50S (50) | 50S (50) |  | 5SMS (4.5) | 30MSS (27) |  | 60S (60) | 45SMS (40.5) |
| 78 | - | 15SMS (13.5) |  | 20MSS (18) | 10MS (8) |  | 30MSMR (18) | 30SMS (27) |
| 79 | 25MRMS (15) | 40MRMS (24) |  | 20MS (16) | 20MS (16) |  | 40MSMR (24) | 30MRMS (18) |
| 80 | 30MS (24) | 40SMS (36) |  | 40MSS (36) | 30MS (24) |  | 50SMS (45) | 30MSS (27) |
| 81 | 30MSS (27) | 30SMS (27) |  | 20MSS (18) | 25MSS (22.5) |  | 50MS (40) | 50SMS (45) |
| 82 | - | 40SMS (36) |  | 30MSS (27) | 40S (40) |  | 50S (50) | 55S (55) |
| 83 | 40MSS (36) | 40SMS (36) |  | 20MS (16) | 20MS (16) |  | 40MSMR (24) | 50MSS (45) |
| 84 | 50SMS (45) | 30MSS (27) |  | 25MSS (22.5) | 30SMS (27) |  | 40SMS (36) | 40S (40) |
| 85 | 60S (60) | 60S (60) |  | 25MSS (22.5) | 25MSS (22.5) |  | 60S (60) | 50S (50) |
| 86 | TSMR (0.2) | 20MR (8) |  | 20MS (16) | 10MS (8) |  | 50SMS (45) | 50SMS (45) |
| 87 | 40SMS (36) | 40MS (32) |  | 10MS (8) | 40MSS (36) |  | 70SMS (63) | 60S (60) |
| 88 | 20MSMR (12) | - |  | 20MSS (18) | 30MSS (27) |  | 40SMS (36) | 30SMS (27) |
| 89 | 40S (40) | 60S (60) |  | 30SMS (27) | 40SMS (36) |  | 60MSMR (36) | 60S (60) |
| 90 | 30S (30) | 40S (40) |  | 50SMS (45) | 50S (50) |  | 50S (50) | 50S (50) |
| 91 | 15MS (12) | 5MRMS (3) |  | 20MSS (18) | 20MS (16) |  | 30MS (24) | 30MSS (27) |
| 92 | 25MSS (22.5) | 20MS (16) |  | 40S (40) | 20MSS (18) |  | 40MSS (36) | 15MS (12) |
| 93 | 5MS (4) | 15SMS (13.5) |  | 40MSS (36) | 40MSS (36) |  | 30SMS (27) | 15MSS (13.5) |
| 94 | 10MSS (9) | 20SMS (18) |  | 10MSS (9) | 30MSS (27) |  | 30SMSMR (18) | 40S (40) |
| 95 | 10MS (8) | 30MSMR (18) |  | 20MSS (18) | 10MS (8) |  | 35MSMR (21) | 20MSMR (12) |
| 96 | 30MRMS (18) | 40MSMS (24) |  | 30SMS (27) | 40SMS (36) |  | 50MSS (45) | 60S (60) |
| 97 | 20MS (16) | 30MR, 5S (12) |  | 40SMS (36) | 30SMS (27) |  | 50SMS (45) | 30MS (24) |
| 98 | 70S (70) | 70S (70) |  | 30MSS (27) | 30MSS (27) |  | 40MS (32) | 70S (70) |
| 99 | 5MS (4) | - |  | 20MSS (18) | 25MS (20) |  | 20MSS (18) | 15SMS (13.5) |
| 100 | 15MSS (13.5) | 15SMS (13.5) |  | 40MSS (36) | 25MS (20) |  | 40S (40) | 50S (50) |
| 101 | - | 15S (15) |  | 30MSS (27) | 30MSS (27) |  | 25MSS (22.5) | 30S (30) |
| 102 | - | 15MSS (13.5) |  | 10MS (8) | 40MSS (36) |  | 30SMS (27) | 30S (30) |
| 103 | - | 10R (2) |  | 5MS (4) | 20MSS (18) |  | 30SMS (27) | 20MS (16) |
| 104 | 30SMS (27) | 30SMS (27) |  | 40MSS (36) | 40MSS (36) |  | 50SMS (45) | 50S (50) |
| 105 | 15MSS (13.5) | - |  | 20MSS (18) | 20S (20) |  | 20MSS (18) | 30S (30) |
| 106 | 0 (0) | - |  | 20MSS (18) | 30MSS (27) |  | 20MS (16) | 20MS (16) |
| 107 | 15MSS (13.5) | 15SMS (13.5) |  | 40MSS (36) | 40S (40) |  | 40SMS (36) | 30MSS (27) |
| 108 | - | 10SMS (9) |  | 25MSS (22.5) | 20MSS (18) |  | 25MRMS (15) | 30S (30) |
| 109 | - | 15MSS (13.5) |  | 40S (40) | 30MS (24) |  | 40MSS (36) | 50SMS (45) |
| 110 | 15MSS (13.5) | 15MS (12) |  | 20SMS (18) | 10SMS (9) |  | 15MS (12) | 25MS (20) |
| 111 | 10MSS (9) | 50SMS (45) |  | 30SMS (27) | 20SMS (18) |  | 5MS (4) | 20MS (16) |
| 112 | 40MSMR (24) | - |  | 20MS (16) | 40S (40) |  | 60SMS (54) | 60S (60) |
| 113 | TRS (0.2) | TMRMS (0.2) |  | 30SMS (27) | 30S (30) |  | 20MSMR (12) | 15MSMR (9) |
| CI 14275 | 5RMR (1.5) | TMRMS (0.2) |  | TMS (0.2) | 5MS (4) |  | 10R-TMS (2) | 15MSMR (9) |
| LMPG-6 | 70S (70) | 70S (70) |  | 50S (50) | 40S (40) |  | 70S (70) | 40S (40) |

^a^ Stem rust severity was visually scored based on the modified Cobb scale of 0-100 (Peterson *et al*., 1948). The responses were given as described by Roelfs *et al.* (1992). Indicated in parentheses are coefficient of infection (COI) values generated by multiplying stem rust severity for each line by a constant value for each infection response: 0 = 0, R = 0.2, RMR= 0.3, MR = 0.4, M = 0.6, MS = 0.8, S = 1.0 (Knott, 1989).
