# Supplementary material for: Mapping and Validation of Stem Rust Resistance Loci in Spring Wheat Line CI 14275
Source: Front Plant Sci. 2021 Jan 12;11:609659. doi: 10.3389/fpls.2020.609659 (PMC7835402; doi:10.3389/fpls.2020.609659)
Supplement: Supplementary Table 5 — Stem rust severity and response of 113 recombinant inbred lines (RILs) of the cross LMPG-6/C I14275 and the parents to US Pgt races in St. Paul, MN, in 2017 (STP17) and 2018 (STP18). Data for the two replicates are displayed. [file Table_5.DOCX]

**Supplementary Table 5.** Stem rust severity and response of 113 recombinant inbred lines (RILs) of the cross LMPG-6/C I14275 and the parents to US *Pgt* races in St. Paul, MN in 2017 (STP17) and in 2018 (STP18). Data for the two replicates are displayed^a^.

|  | STP17 | |  | STP18 | |
| --- | --- | --- | --- | --- | --- |
| Line Number/Name | Rep I | Rep II |  | Rep I | Rep II |
| 1 | 70S (70) | 70S (70) |  | 100S (100) | 100S (100) |
| 2 | 10MR (4) | 20MRMS (12) |  | 10RMR (3) | 15RMR (4.5) |
| 3 | 60S (60) | 40S (40) |  | 50S (50) | 40M (24) |
| 4 | 60S (60) | 90S (90) |  | 100S (100) | 100S (100) |
| 5 | 25MSMR (15) | 20MSMR (12) |  | 10M (6) | 15M (9) |
| 6 | 20MSMR (12) | 20MRMS (12) |  | 15RMR (4.5) | 20M (12) |
| 7 | 15MRMS (9) | 15MRMS (9) |  | 1RMR (0.3) | 15RMR (4.5) |
| 8 | 70S (70) | 50S (50) |  | 80S (80) | 90S (90) |
| 9 | 20MSMR (12) | 40MSMR (24) |  | 40M (24) | 50S (50) |
| 10 | 20MRMS (12) | 15MRMS (9) |  | 15M (9) | 10RMR (3) |
| 11 | 30MSS (27) | 40S (40) |  | 60S (60) | 60S (60) |
| 12 | 40MSMR (24) | 40MSMR (24) |  | 60S (60) | 40M (24) |
| 13 | 70S (70) | 50MS (40) |  | 60S (60) | 90S (90) |
| 14 | 20MRMS (12) | 25MRMS 15) |  | 40M (24) | 30M (18) |
| 15 | 70S (70) | 70S (70) |  | 100S (100) | 100S (100) |
| 16 | 90S (90) | 70S (70) |  | 100S (100) | 100S (100) |
| 17 | 60MSMR (36) | 25MRMS 15) |  | 100S (100) | 90S (90) |
| 18 | 15MSS (13.5) | 40S (40) |  | 60S (60) | 40MS (32) |
| 19 | 70S (70) | 60S (60) |  | 100S (100) | 100S (100) |
| 20 | 60S (60) | 60S (60) |  | 50S (50) | 60S (60) |
| 21 | 80S (80) | 90S (90) |  | 100S (100) | 100S (100) |
| 22 | 15MRMS (9) | 25MRMS (15) |  | 5RMR (1.5) | 30M (18) |
| 23 | 5RMR (1.5) | 5MS (4) |  | TR (1) | 10M (6) |
| 24 | 60S (60) | 60S (60) |  | 80S (80) | 60S (60) |
| 25 | 70S (70) | 80S (80) |  | 80S (80) | 100S (100) |
| 26 | 15MS (12) | 40MSMR (24) |  | - | - |
| 27 | 50MRMS (30) | 20MRMS (12) |  | 10RMR (3) | 25M (15) |
| 28 | 30MSS (27) | 40MS (32) |  | 25MSS (22.5) | 50S (50) |
| 29 | 5RMR (1.5) | 20MSMR (12) |  | 15M (9) | 30MSS (27) |
| 30 | 90S (90) | 70S (70) |  | 100S (100) | 100S (100) |
| 31 | 70S (70) | 50S (50) |  | 100S (100) | 80S (80) |
| 32 | 30MRMS (18) | 15MRMS (9) |  | 30M (18) | 30M (18) |
| 33 | 40MSS (36) | 30MSMR (18) |  | 60S (60) | 60S (60) |
| 34 | 50S (50) | 70S (70) |  | 80S (80) | 70S (70) |
| 35 | 5MS (4) | 5RMR (1.5) |  | 5M (3) | 5RMR (1.5) |
| 36 | 70S (70) | 60S (60) |  | 80S (80) | 90S (90) |
| 37 | 25MSMR (15) | 40MSMR (24) |  | 15M (9) | 25M (15) |
| 38 | 25MRMS (15) | 20MRMS (12) |  | 30M (18) | 25M (15) |
| 39 | 70S (70) | 70S (70) |  | 100S (100) | 100S (100) |
| 40 | 60S (60) | 50S (50) |  | 100S (100) | 90S (90) |
| 41 | 40MS (32) | 40MRMS (24) |  | 60S (60) | 20M (12) |
| 42 | 60S (60) | 90S (90) |  | 100S (100) | 100S (100) |
| 43 | 30MSS (27) | 30MS (24) |  | 50S (50) | 30MS (24) |
| 44 | 90S (90) | 80S (80) |  | 100S (100) | 100S (100) |
| 45 | 25MSS (22.5) | 40MS (32) |  | 50S (50) | 60S (60) |
| 46 | 15MS (12) | 15MS (12) |  | 15M (9) | 30MS (24) |
| 47 | 70S (70) | 50S (50) |  | 100S (100) | 100S (100) |
| 48 | 30MSMR (18) | 50MS (40) |  | 30M (18) | 20M (12) |
| 49 | 60S (60) | 50S (50) |  | 60S (60) | 50S (50) |
| 50 | 40MSMR (24) | 50MRMS (30) |  | 60S (60) | 70S (70) |
| 51 | 60S (60) | 40MSMR (24) |  | 100S (100) | 100S (100) |
| 52 | 40MSMR (24) | 20MS (16) |  | 40MSS (36) | 30M (18) |
| 53 | 15MRMS (9) | 20MRMS (12) |  | 10RMR (3) | 15M (9) |
| 54 | 70S (70) | 50S (50) |  | 50S (50) | 50S (50) |
| 55 | 70S (70) | 40MSMR (24) |  | 100S (100) | 100S (100) |
| 56 | 25MSMR (15) | 40MS (32) |  | 60S (60) | 40S (40) |
| 57 | 50MS (40) | 50MSMR (30) |  | 80S (80) | 100S (100) |
| 58 | 50MSMR (30) | 30MSMR (18) |  | 20M (12) | 30M (18) |
| 59 | 80S (80) | 40MS (32) |  | 100S (100) | 100S (100) |
| 60 | 10RMR (3) | 10MR (4) |  | 1RMR (0.3) | 15RMR (4.5) |
| 61 | 40MRMS (24) | 40MRMS (24) |  | 50S (50) | 30M (18) |
| 62 | 25MSMR (15) | 15MRMS (9) |  | 15M (9) | 25M (15) |
| 63 | 80S (80) | 60S (60) |  | 100S (100) | 100S (100) |
| 64 | 25MS (20) | 40S (40) |  | 25MSS (22.5) | 50S (50) |
| 65 | 25MSMR (15) | 40MSMR (24) |  | 10RMR (3) | 15M (9) |
| 66 | 40MS (32) | 40MS (32) |  | 50S (50) | 40M (24) |
| 67 | 25MSS (22.5) | 15M (9) |  | 15M (9) | 30MSS (27) |
| 68 | 60S (60) | 40S (40) |  | 70S (70) | 80S (80) |
| 69 | 60S (60) | 50MSMR (30) |  | 90S (90) | 80S (80) |
| 70 | 80S (80) | 50MS (40) |  | 100S (100) | 100S (100) |
| 71 | 10MRMS (6) | 15MS (12) |  | 15M (9) | 15M (9) |
| 72 | 20MRMS (12) | 30MRMS (18) |  | 60S (60) | 40M (24) |
| 73 | 50MSMR (30) | 60S (60) |  | 70S (70) | 100S (100) |
| 74 | 70S (70) | 50S (50) |  | 60S (60) | 50S (50) |
| 75 | 50S (50) | 50S (50) |  | 90S (90) | 50S (50) |
| 76 | 50MS (40) | 40MS (32) |  | 30M (18) | 50S (50) |
| 77 | 60S (60) | 70S (70) |  | 80S (80) | 100S (100) |
| 78 | 15MRMS (9) | 5RMR (1.5) |  | 30M (18) | 30M (18) |
| 79 | 15MRMS (9) | 40MRMS (24) |  | 25M (15) | 25M (15) |
| 80 | 30MSMR (18) | 40MSMR (24) |  | 40M (24) | 50S (50) |
| 81 | 15MSMR (9) | 15MRMS (9) |  | 10RMR (3) | 15RMR (4.5) |
| 82 | 80S (80) | 50S (50) |  | 70S (70) | 100S (100) |
| 83 | 25MRMS (15) | 15MRMS (9) |  | 50S (50) | 40M (24) |
| 84 | 50MRMS (30) | 30MSMR (18) |  | 30M (18) | 30M (18) |
| 85 | 50MSMR (30) | 40MRMS (24) |  | 80S (80) | 50S (50) |
| 86 | 25MSMR (15) | 40MSMR (24) |  | 90S (90) | 50M (30) |
| 87 | 15MS (12) | 40S (40) |  | 50S (50) | 30MSS (27) |
| 88 | 25MSMR (15) | 30MSMR (18) |  | 15M (9) | 60S (60) |
| 89 | 70S (70) | 90S (90) |  | 40M (24) | 100S (100) |
| 90 | 80S (80) | 80S (80) |  | 100S (100) | 100S (100) |
| 91 | 50MSMR (30) | 25MSMR (15) |  | 50S (50) | 50S (50) |
| 92 | 50S (50) | 25MSMR (15) |  | 25M (15) | 15M (9) |
| 93 | 30MS (24) | 25MS (20) |  | 25MS (20) | 15MSS (13.5) |
| 94 | 20MRMS (12) | 30MRMS (18) |  | 15M (9) | 15RMR (4.5) |
| 95 | 60S (60) | 40MS (32) |  | 25M (15) | 40M (24) |
| 96 | 40MSMR (24) | 40MSMR (24) |  | 30M (18) | 50M (30) |
| 97 | 30MS (24) | 40S (40) |  | 30M (18) | 40M (24) |
| 98 | 80S (80) | 90S (90) |  | 100S (100) | 100S (100) |
| 99 | 15MS (12) | 30S (30) |  | 15M (9) | 40MS (32) |
| 100 | 70S (70) | 60S (60) |  | 50S (50) | 50S (50) |
| 101 | 5RMR (1.5) | 5MS (4) |  | 20M (12) | 15MSS (13.5) |
| 102 | 30S (30) | 40S (40) |  | 60S (60) | 50S (50) |
| 103 | 10MRMS (6) | 10MRMS (6) |  | 10RMR (3) | 25M (15) |
| 104 | 40MS (32) | 20MS (16) |  | 50S (50) | 50S (50) |
| 105 | 50S (50) | 60S (60) |  | 30M (18) | 30MSS (27) |
| 106 | 5RMR (1.5) | 5RMR (1.5) |  | 5RMR (1.5) | 5RMR (1.5) |
| 107 | 15MS (12) | 15MS (12) |  | 10M (6) | 25M (15) |
| 108 | 50MS (40) | 25MSMR (15) |  | 40M (24) | 40M (24) |
| 109 | 70MSS (63) | 50S (50) |  | 50S (50) | 60S (60) |
| 110 | 15MSS (13.5) | 15MS (12) |  | 5RMR (1.5) | 5RMR (1.5) |
| 111 | 5MS (4) | 15MS (12) |  | 15M (9) | 15M (9) |
| 112 | 15MS (12) | 25MS (20) |  | 15M (9) | 25M (15) |
| 113 | 60S (60) | 40M (24) |  | 40M (24) | 25M (15) |
| CI 14275 | 5RMR (1.5) | 5RMR (1.5) |  | 0 (0) | TR (1) |
| LMPG-6 | 100S (100) | 90S (90) |  | 90S (90) | 100S(100) |

^a^ Stem rust severity was visually scored based on the modified Cobb scale of 0-100 (Peterson *et al*., 1948). The responses were given as described by Roelfs *et al.* (1992). Indicated in parenthesis are coefficient of infection (COI) values generated by multiplying stem rust severity for each line by a constant value for each infection response: 0 = 0, R = 0.2, RMR= 0.3, MR = 0.4, M = 0.6, MS = 0.8, S = 1.0 (Knott, 1989).
